# Isolation and characterization of phage ISTP3 for bio-control application against drug-resistant *Salmonella*

**DOI:** 10.3389/fmicb.2023.1260181

**Published:** 2023-11-22

**Authors:** Md. Sharifull Islam, Ishatur Nime, Fan Pan, Xiaohong Wang

**Affiliations:** ^1^Key Laboratory of Environment Correlative Dietology, College of Food Science and Technology, Huazhong Agricultural University, Wuhan, China; ^2^Center for Cancer Immunology, Institute of Biomedicine and Biotechnology, Shenzhen Institute of Advanced Technology, Chinese Academy of Sciences, Shenzhen, China

**Keywords:** phage, drug-resistant; *salmonella*, characterization, biological control, foods

## Abstract

*Salmonella* including drug-resistant strains are major foodborne pathogens causing serious illness and pose a great threat to the prevention and control for food safety. Phages can naturally defect the bacterium, is considered as a new and promising biological antimicrobial agent in the post-antibiotic era. A poultry facility in Wuhan, China provided wastewater samples from which a collection of 29 phages were isolated and purified. A broad host spectrum phage ISTP3, which capable of infecting all tested *Salmonella*, including drug-resistant *Salmonella enterica*, were examined. Additionally, the effectiveness of this phage ISTP3 in reducing drug-resistant *S. enterica* was assessed in diverse food samples. Transmission electron microscopy (TEM) and whole genome sequencing demonstrated that ISTP3 was found to belong to family *Ackermannviridae*. The one-step growth experiment and assays of stability demonstrated that ISTP3 exhibited short periods of inactivity before replicating, produced a significant number of viral progeny during infection, and remained high stable under varying pH and temperature conditions. We evaluated the efficacy of phage ISTP3 against drug-resistant *Salmonella* on chicken breast and lettuce samples at different temperatures. When applying phage ISTP3 in food matrices, the drug resistant *Salmonella* count significantly reduced at 4°C and 25°C at an MOI of 100 or 1,000 within a timescale of 12 h. Overall, the results, such as broad host ranges, strictly lytic lifestyles, absence of lysogenic related genes, toxin genes, or virulence genes in the genome, demonstrate that the application of phage ISTP3 as a biocontrol agent has promising potential for preventing and controlling drug-resistant *S. typhimurium* in the context of food safety, processing, and production.

## 1 Introduction

*Salmonella* bacteria, which are members of the *Enterobacteriaceae* family, have a rod-shaped morphology with a diameter ranging from 0.2 to 1.5 μm, and they are facultative anaerobes. They can be classified as gram-negative bacilli (Ali et al., 2018). *Salmonella* genus can be classified into two main species, *S. enterica* and *S. bongori*, which are distinguished by variations in their 16S rRNA sequence. *S. enterica* encompasses a total of 2,600 distinct serovars and a many of these are responsible to cause diseases for humans and animals (Yang et al., 2019). In the world *Salmonella* is described as the most common foodborne pathogen, mainly infect to humans through contaminated food matrices (Jiang et al., 2021). Infected animals and their faeces act as reservoirs for the contamination of *Salmonella* and this contamination can then spread through products like eggs, meat, milk, and other agricultural goods that have been fertilized or grown in feces contaminated with *Salmonella* (Finn et al., 2013). It is estimated that globally approximately 500,000 deaths are caused by 33 million *Salmonella* infections (Zhou et al., 2023). While *Salmonella* infection is highly suspected in age groups <20 years and >70 years, the highest incidence is observed in infants, with 130 cases per 100,000 individuals (Zaki and Karande, 2011). Approximately 25 to 33% of children diagnosed with typhoid fever are below the age of 5, with a subset of 6 to 21% being younger than 2 years old (Owais et al., 2010). A group of scientist from USA reported that the chance of hospitalization due to 47–80% for infected people aged ≥65 years because of non-typhoidal *Salmonella* infection (Chen et al., 2012).

Freshly prepared vegetables provide an enjoyable way to incorporate essential dietary fiber, vitamins, and minerals which are vital components of a healthy diet. Hence, these globally renowned food items pose a significant risk to public health if consumed raw or subjected to insufficient heat treatment, as their fresh form may potentially contain harmful bacterial contaminants (Pem and Jeewon, 2015; Moye et al., 2018). In the past 15 years during the years of 1990 to 2005, there were 768 outbreaks associated with fresh produce, leading to 35,060 reported cases of illness (Sharma, 2013). Lettuce, a highly popular leafy vegetable worldwide, is typically consumed raw and has been frequently associated with outbreaks of foodborne diseases such as salmonellosis. The prevalence of *Salmonella* in lettuce was found to be 0.064 in developing countries and 0.028 in developed countries, respectively (De Oliveira Elias et al., 2019). In China, the prevalence of *Salmonella* in raw vegetables was found to be 3.4% (14 out of 406 samples). Among the different vegetables tested, coriander exhibited the highest contamination rate at 7.8% (90 out of the samples), followed by lettuce at 6.0% (83 out of the samples) (Yang et al., 2020). Furthermore, *Salmonella* is commonly present in the intestinal tract of warm-blooded animals, particularly in chickens. This facilitates the easy transfer of *Salmonella* to meat during processes such as slaughter, handling, processing, and distribution. Chicken meat has been identified as a significant source of *Salmonella* infections in humans, contributing to global outbreaks, including those involving drug-resistant strains (Mori et al., 2018; Duc et al., 2019; Wessels et al., 2021). In 2019, an extensive investigation was carried out on 3,508 samples collected from poultry breeding farms across nine provinces in China. The study identified 126 strains of *Salmonella*, indicating a positivity rate of 3.59% for the samples (Song et al., 2020). During the period from April to November 2011, a study conducted by Alali et al. (2012) reported a *Salmonella* prevalence of 31.5% (*n* = 698) in chicken samples collected from Russia. In Malaysia the prevalence of *Salmonella* was found 39.4% (*n* = 156) in the year of 2017 (Thung et al., 2017). *Salmonella* was responsible for over 50% of the reported cases of foodborne illnesses in the European Union (Ehuwa et al., 2021). The economic impact of foodborne *Salmonella* in the United States has been substantial. According to the USDA’s Economic Research Service (ERS), the total cost for foodborne *Salmonella* infections in the United States was estimated at $4.1 billion annually (Ford et al., 2023).

The estimated annual cost for *Salmonella* control program is increasing in some countries (Niemi et al., 2019). Various industrialized systems have been developed for addressing bacterial contamination, including *Salmonella*, in food. However, these methods still have specific disadvantages, such as potential harm or toxicity to food, inconsistency in quality, and high practical application costs (Muncke et al., 2020; Lebelo et al., 2021; Wessels et al., 2021). One instance is the use of non-thermal methods like washing food in chlorinated water, which fails to completely eliminate enteric bacterial pathogens. This is primarily due to challenges such as biofilm formation, inaccessible attachment sites, the strength of attachment, or internalization of the pathogen (Gil Prieto et al., 2009). However, the use of chemicals has also led to the emergence of drug-resistant bacteria, posing a significant public health risk (Racine and Gualtieri, 2019). Drug-resistant poses a major threat to global human health, as it makes the treatment of pathogen infections challenging due to their high prevalence rate, associated mortality, and treatment costs. The potential transmission link to humans from antibiotic-resistant *Salmonella* at the farm level can occur through direct contact or consumption of contaminated meat (Dolejska et al., 2012). Globally, antimicrobial-resistant *Salmonella* strains have become increasingly prevalent over the past decade (Pelyuntha et al., 2021). Therefore, it is imperative to search for control agents that enable the production of food with minimal or no harm to human consumption, without compromising the organoleptic and nutritional quality of the food.

Bacteriophages, also known as phages, are naturally existing viruses that vary in size from 20 to 200 nm and act as predators of bacteria (D'accolti and Soffritti, 2021). Phages, which are present everywhere in the environment, pose no harm to humans and animals. Phages have gained recognition as promising antimicrobial agents that can assist in controlling specific bacterial pathogens in food processing or production, potentially saving millions of lives and revolutionizing the field of medicine since their discovery (Hyla et al., 2022). On the contrary, phages are considered one of the most crucial, cost-effective, and straightforward approaches to combat microbes in both planktonic and biofilm environments (Totten and Patel, 2022). Consequently, lytic phages are increasingly being employed as alternative tools to eliminate pathogenic bacteria in food products and combat drug-resistant bacteria. Due to their ability to offer a safe, natural, and effective solution against pathogenic bacterial contamination in fresh produce items and chicken meat, various national and international regulatory authorities are progressively granting approval for the utilization of phages (García et al., 2019). The application of targeted phage treatment in the food industry can prevent product degradation and the transmission of bacterial diseases. As a result, this will ultimately enhance safety in the production, processing, and handling of animal and plant-based food, creating safe environments. From a food safety standpoint, phages have been suggested as a novel, efficient, stable, and safe antibacterial method, considered one of the least harmful approaches (Lee et al., 2022). In this study, we isolated, screened, and fully characterized phage ISTP3, which demonstrated sustained activity in reducing drug-resistant *S. enterica* specifically in food applications, particularly for lettuce and chicken meat.

## 2 Materials and methods

### 2.1 Bacteria strains and growth conditions

Drug-resistant *S. typhimurium* SA32 was used as a host for isolation of phage. For the host range activity, we employed a collection of 43 distinct bacterial strains, including 31 strains of *Salmonella* and 12 strains of bacteria that were not of the *Salmonella* type (Table 1). All bacterial strains were properly preserved at −80°C with a 20% (v/v) glycerol solution. Prior to the experiment, the strains were cultured on Luria-Bertani agar (LA) agar plates at 37°C using the streak plate method. A single colony was selected and inoculated into Luria-Bertani broth (LB) broth medium at 37°C overnight to validate the purification of the bacteria.

**Table 1:** Host range of phage ISTP3, ISTP6, ISTP8, ISTP14, ISTP18, ISTP20, and ISTP26 against different *Salmonella* serovars and other bacterial strains determined by spot testing.

Bacterial strains	Drug-resistant	ISTP3	ISTP6	ISTP8	ISTP14	ISTP18	ISTP20	ISTP26
*S. enterica* serovar Typhimurium								
ATCC 14028	/	++++	+++	+	+	++	++	+++
ATCC 13311	/	++++	++++	+	+++	+	+++	+
UK-1	/	++++	+	+	+	+++	+	+
ST8	/	++++	++	+++	+	++	+	+++
SGSC 4903	/	++++	+	++	+	+++	+	+
SL 1344	/	++++	++	+++	+	++	+++	+++
LT2	/	++++	+	++	+	+++	+	+
*S. enterica* serovar Enteritidis								
ATCC 13076	/	++++	+	+	+	+	+	+
SJTUF 10978	/	++++	+	+++	+	++	++	+++
SJTUF 10984	/	++++	+	+	+	+	++	+
LK5-3820	/	++++	+	+	+	++	++	+++
SGSC 4901	/	++++	++	+	+	+	++	+
*S. enterica* serovar Dublin								
3,710	/	++++	−	−	−	−	−	+
3,723	/	++++		−	+	+	−	−
*S. enterica* serovar Newport								
E20002725	/	+++	−	+	−	+	−	−
*S. enterica* serovar Paratyphi B								
CMCC 50094	/	++++	++	−	+	+	−	+
*S. enterica* Serovar Pullorum								
CVCC 519	/	++++	−	−	−	−	−	+
*S. enterica* subsp. *enterica* serovar Javiana								
CVM 35943	/	++++	−	+	−	+	−	+
*S. enterica* subsp. *enterica* serovar Anatum								
ATCC 9270	/	++++	−	+	−	+	−	−
*S. enterica* subsp. *enterica* serovar Kentucky								
CVM 29188	/	++++	−	−	−	−	+	−
*S. enterica* subsp. *arizonae*								
CDC346-86	/	++++	+	−	−	−	+	−
Drug-resistance *Salmonella*								
*S. enterica* serovar Typhimurium								
SA32 AMP/STR/GEN/KAN/CHL/CIP/SUL/SXT		++++	++	+	+	+	+	+
SA33 AMP/STR/GEN/KAN/CHL/CIP/SUL/SXT		++++	+	++	−	+	++	+
SA34 AMP/STR/GEN/KAN/CHL/CIP/SUL/SXT		++++	+	+	+	+	+	++
SA35 AMP/STR/GEN/KAN/CHL/CIP/SUL/SXT		++++	++	++	+	+	++	+++
SA36 AMP/STR/GEN/KAN/CHL/CIP/SUL/SXT		++++	+	++	+++	++	++	+
*S. enterica* serovar Enteritidis			+					
SA29 LVX/AMP/SXT		++++	+	+	+	−	+	+
SA30 CIP/LVX/AMP/SXT		++++	+	+	+	+	+++	+
*S. enterica* serovar Indiana								
SA27 CIP/LVX/AMP/SXT		++	+	+	+	++	++	+
SA28 CIP/LVX/AMP/SXT		++	+	+	+	+	+	+
*S. enterica* serovar Typhi								
SA31	CIP	++++	++	+	+	+	++	+
Other Gram-positive bacteria								
*Staphylococcus aureus* ATCC 6538		−	−	−	−	−	−	−
*Staphylococcus aureus* ATCC 29213		−	−	−	−	−	−	−
*Listeria monocytogenes* ATCC 19115		−	−	−	−	−	−	−
*Lactobacillus acidophilus* ATCC SD5221		−	−	−	−	−	−	−
Gram-negative bacteria								
*Escherichia coli* BL21		−	−	−	−	−	−	−
*Escherichia coli* DH5α		−	−	−	−	−	−	−
*Escherichia coli* T10		−	−	−	−	−	−	−
*Escherichia coli* C83715		−	−	−	−	−	−	−
*Shigella flexneri* CMCC 51572		−	−	−	−	−	−	−
*Vibrio parahaemolyticus* ATCC 17802		−	−	−	−	−	−	−
*Vibrio parahaemolyticus* ATCC 33846		−	−	−	−	−	−	−
*Pseudomonas aeruginosa* ATCC 7853		−	−	−	−	−	−	−

++++, complete clearing; +++, clearing throughout but with faintly hazy background; ++, substantial turbidity throughout the cleared zone; +, a few in individual plaques; –, no clearing. AMP, Ampicillin; CIP, Ciprofloxacin; LVX, Levofloxacin; STR, Streptomycin; GEN, Gentamicin; KAN, Kanamycin; CHL, Chloramphenicol; SUL, sulfonamide; and SXT, Sulfamethoxazole.

### 2.2 Isolation, propagation, and purification of phage

According to established protocols (Islam et al., 2021), a collection of 29 putative distinct phages were collected from wastewater samples taken from a chicken farm located in Wuhan, China. The enrichment process for phage isolation was altered based on a previous study conducted by Guo et al. (2021). A mixture was created by combining 5 mL of a sample filtered through a 0.22 μm filter with 10 mL of LB and 2.5 mL of the host bacterial strain. The mixture was left to incubate overnight at a temperature of 37°C. After the incubation period, the culture was collected and subjected to centrifugation at 11,200 × g for 10 min at 4°C. The resulting supernatants were then filtered using a 0.22 μm pore size filter. The phage lysate, which is the liquid obtained after removing the sediment, was collected and utilized in spot tests to identify the presence of phages. To purify the phage plaques, a single plaque was chosen and cultured in 1 mL of LB medium along with the host bacteria. The propagation process was repeated at least four times to ensure the purification of the phage as a single entity. The purified phages were stored at a temperature of 4°C until they were needed for various experiments.

### 2.3 Spot test

The phages capability for lysis diverse strains was identified using spot test. In brief, 100 μL of target exponential growth phase cultured bacteria with 4 mL 0.7% agar was poured on LA plates then mixed and wait for 15 min at room temperature to solidify. Subsequently, 5 μL of phage lysates were spotted onto the lawns formed by different bacterial strains. The Petri dishes were then incubated at 37°C for 24 h. Following incubation, the formation of clear zones on the bacterial lawn was determined as an indication of phage sensitivity.

### 2.4 Efficiency of plating

To assess the host range, we performed an Efficiency of Plating (EOP) assay with modifications based on previous studies (Islam et al., 2019b). The phage was serially diluted and triplicates were tested on the designated bacterial host. The bacterial strains were cultivated to the exponential phase at 37°C. After incubation, 100 μL of the bacterial culture was applied in double-layer plate assays along with 100 μL of the diluted phage lysate. Dilution factors ranging from 10^6^ to 10^9^ were applied in this study. Subsequently, the plates were incubated at 37°C for 12 h, and the number of plaque-forming units (PFUs) on the plate was enumerated. The relative EOP was then calculated by dividing the average PFUs on the test bacteria by the average PFUs on the host bacteria. The EOP assessment was categorized as follows: high efficiency (EOP 0.5 to 1.0), moderate efficiency (EOP 0.1 to 0.49), low efficiency (0.001 to 0.9), and inefficient (0 to 0.009).

### 2.5 Transmission electron microscopy

Freshly prepared phage suspension (10^10^ PFU/mL) was re-suspended in SM buffer after ultracentrifugation at 50,000 × g for 2 h at 4°C. Negative staining method (2% phosphotungstic acid) was used for preparation of phages to observe their morphology under transmission electron microscopy. The TEM images were analyzed using the software Digital Micrograph Demo 3.9.1.^1^

### 2.6 Absorption rate

Assays for phage adsorption were conducted following the methodology previously described by Fister et al. (2016), with slight modifications. In brief, a fresh phage solution and a host bacterial solution were combined in a sterile tube at an optimal MOI value (MOI 0.01), followed by incubation at 37°C for 20 min on a shaker. The mixture was subsequently centrifuged at 12,000 × g for 5 min, and the resulting supernatants were immediately filtered using 0.22 μm filters (Millipore, Ireland). The filtered supernatants, which contained unabsorbed phages, were then quantified using a double-layered agar plate method. The adsorption percentage was calculated by comparing the phage titer in the supernatant with control samples with no bacteria.

### 2.7 One-step growth curves of phage ISTP3

The methods used for the one-step growth tests were based on Park et al.’s study (Park et al., 2012) with minor modifications. Briefly, a phage suspension containing 4 log PFU/mL of 500 μL was combined with a host bacterial culture containing 6 log CFU/mL of 500 μL. The mixture was then incubated at 37°C for 10 min to allow phage adsorption to the host, followed by centrifugation at 8000 × g for 5 min. The supernatant was discarded to remove free phage, and the phage attached to the bacterial pellet was gently washed three times with LB. The pellet was subsequently resuspended in 5 mL of LB and cultured at 37°C for 180 min in a shaker at 150 rpm. Two sets of samples were prepared, each with a different duration. In the second set, 1% chloroform was added to the samples before titration to effectively lyse the host bacteria and precisely determine the initial infectious phage count for detecting the eclipse time. The one-step curve was determined by evaluating the phage titers using the double-layered agar plate method for each set of samples.

### 2.8 Thermal and pH stability of phage

To assess the thermal and pH stability of the phage, a method similar to previous studies (Zhang et al., 2023) was employed with some minor modifications. In order to evaluate the thermal stability, the LB medium was heated beforehand, and various temperatures ranging from 30°C to 80°C were maintained for 1 h. Subsequently, the phage lysate (at a concentration of 10^8^ PFU/mL) was mixed into the preheated medium and incubated for 30 and 60 min, respectively. To determine the pH stability of phage ISTP3, the phage lysate (10^8^ PFU/mL) was mixed with LB medium adjusted to different pH levels ranging from 2 to 13, followed by incubation at 37°C for 1 h. At the end of the incubation period, the number of PFU/mL recovered from the test samples was recorded.

### 2.9 Phage sequencing, genome annotation, and comparison

The DNA of phage ISTP3 was isolated and purified based on a previous study conducted by Islam et al. (2020a). Sequencing of ISTP3 was performed using the HiSeq platform from Illumina. The resulting reads were assembled using MicrobeTrakr plus (v0.9.1) software. To predict the open reading frames (ORFs) of ISTP3, three website tools, namely RAST, GeneMarkS, and ORF finder, were applied. Gene annotation was carried out using BLASTP, Pfam, and CD searching against the NCBI nonredundant database and the conserved domain database (CDD). To identify antimicrobial resistant genes (ARGs) within the phage genome, the ResFinder 3.1 database from CGE^2^ was used. Additionally, the VFDB database^3^ was used to identify any potential virulence factors present in the phage genome. The sequences of various phages were compared for whole genome similarities using BLASTN analysis at NCBI. A comparative circular genome map of the phage genome was created using the BRIG comparison tool. The terminase large subunit and the main capsid protein sequence of ISTP3 were compared with the corresponding sequences of related phages using MEGA7. The resulting tree was then enhanced using ITOL.

### 2.10 Biological control of drug resistant *S. enterica* in foods

Iceberg lettuce (*Lactuca sativa*) and raw chicken breast were purchased from a grocery store located at Huzuan Agriculture University in Wuhan, China. The plastic packaging of the lettuce heads was thoroughly rinsed with 70% ethanol before being removed. Outer leaves of the lettuce were discarded, and using a sterile stainless-steel scalpel, inner leaves were cut. Subsequently, 2 cm × 2 cm pieces of lettuce were cut on a sterile wooden tray. The outer surface of chicken breast samples was pull out by the sterilized knife, then cut meat into 2 cm^3^ cubes. Lettuce and chicken samples were confirming any bacteria absence before going for biocontrol test procedure inoculating on LA agar (Guo et al., 2021). Then all the samples were added by 10 μL of drug-resistant *S. typhimurium* SA32 the final concentration 4 log_10_ CFU/sample and keep at room temperature in the laminar air flow hood for 10 min for bacteria to attach on the surface of food. Then, 10 μL of the phage aliquots were added using a micropipette at a final concentration of 6 log (MOI 100) and 7 log (MOI 1000) PFU/sample. The control groups consisted of only bacteria and PBS administration. All samples were incubated for 12 h at either 4°C or 25°C. After the incubation period, bacterial samples were processed for enumeration at 1, 3, 6, and 12 h. All experiments were conducted in triplicate.

### 2.11 Statistical analysis

The food model assays were conducted in triplicates, with two samples per treatment analyzed in each replicate. The mean values of the three replicates were reported, along with error bars indicating the standard deviation of the mean. Both bacterial and phage data were logarithmically transformed. To assess the effectiveness of phage treatment in reducing the number of viable drug-resistant *Salmonella* in the tested foods, the data from the phage treated samples were compared to the control samples treated with PBS. Statistical analysis was performed using Prism 9 for Windows (GraphPad software, San Diego, CA, United States), utilizing a two-way analysis of variance (ANOVA) followed by Bonferroni’s test with a 95% confidence interval. Statistical significance was determined at a significance level of *p* < 0.05.

## 3 Results

### 3.1 Phage isolation and characterization

**Figure 1:**
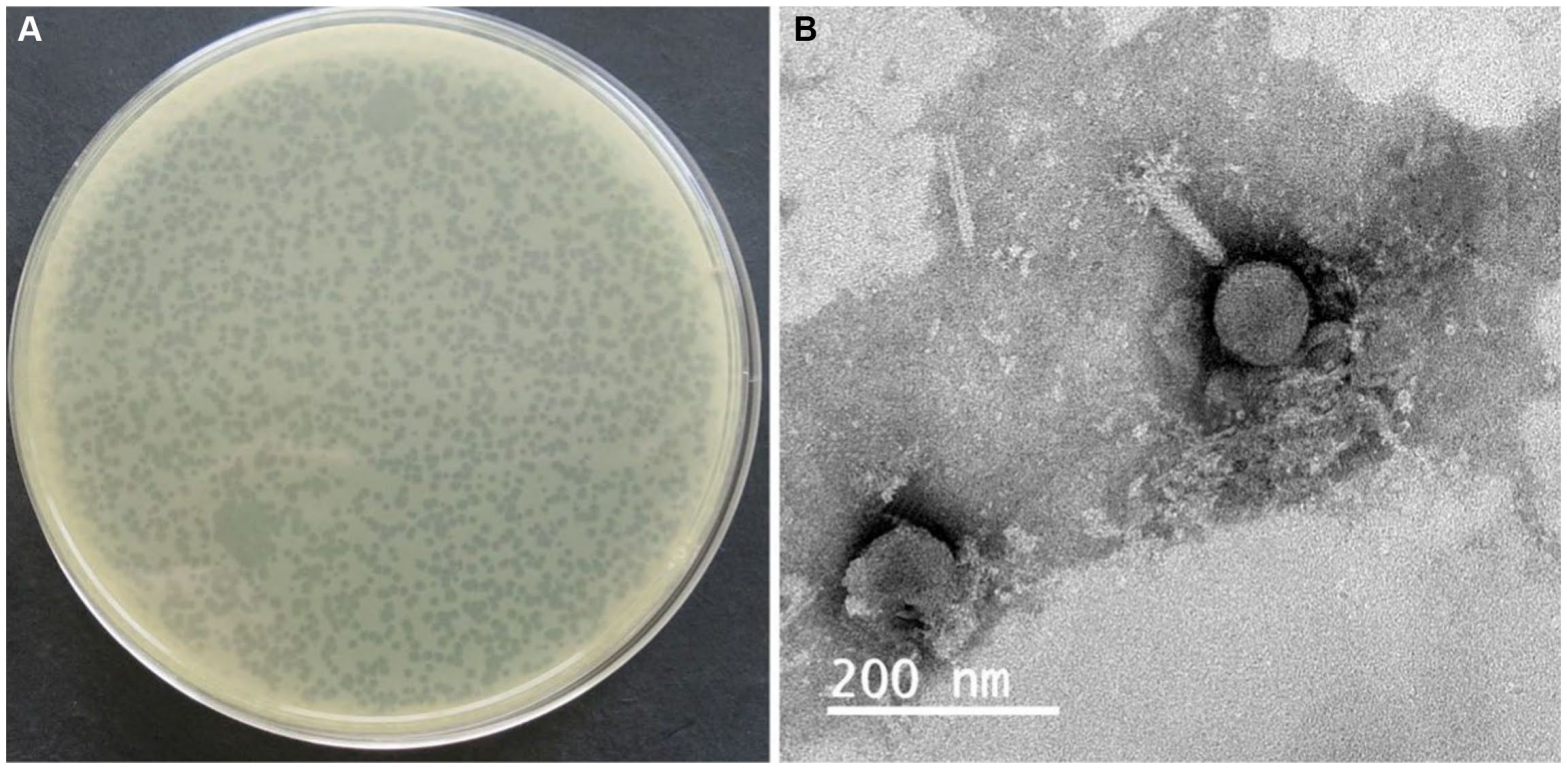
Plaque morphology **(A)** and electron micrographs of phage ISTP3 **(B)**. The bars represent 200 nm.

A total of 29 phages were obtained from wastewater samples taken from a Chinese chicken farm, using drug-resistant *S. typhimurium* SA32 as the host bacterium. All the phages were subsequently tested to determine their efficacy in lysing the different *Salmonella* strains including drug-resistant strains. The results of host range showed that, among those phages, 7 out of 29 is about 24% of the isolates were able to lyse 100% of the *Salmonella* strains tested in this study (Table 2), while the rest were capable to lyse <80% tested strains. Among of them, one phage which showed high lytic activity against different *Salmonella* were selected and named as ISTP3 (Table 1). Spot tests results showed that phage ISTP3 had the highest host range compared to others 6 phages, this specific phage has demonstrated the ability to lyse every tested *Salmonella* strain in the study, encompassing a comprehensive representation. Nevertheless, none of the isolated phages exhibited the ability to cause the lysis of *E. coli* or any other tested gram-positive and gram-negative bacteria. Transmission electron microscope revealed that ISTP3 possessed an icosahedral head with 77 ± 3 nm in diameter, and accompanied by a contractile tail with a 112 ± 4 nm long (Figure 1B). The morphology suggested that ISTP3 belonged to family *Myoviridae*. Host range of the phage was studied using diverse *Salmonella* strains collected by our research group.

**Table 2:** Sensitivity of different *Salmonella* serovars and other bacterial strains to lyse by selected phages determined by spot testing.

Phages	% of positive spot test against drug-resistant *Salmonella* serovars, *Salmonella* serovars Typhimurium and *Salmonella* serovars Enteritidis		
	Drug-resistant *Salmonella* (*N* = 10)	*S. typhimurium* (*N* = 7)	*S. enteritidis* (*N* = 5)
ISTP1	50	57.1	40
ISTP2	40	71.4	60
ISTP3	100	100	100
ISTP4	60	42.8	40
ISTP5	70	71.4	60
ISTP6	100	100	100
ISTP7	50	71.4	60
ISTP8	100	100	100
ISTP9	30	85.7	60
ISTP10	60	42.8	80
ISTP11	70	71.4	40
ISTP12	50	57.4	40
ISTP13	40	85.7	40
ISTP14	100	100	100
ISTP15	60	85.7	20
ISTP16	50	71.4	20
ISTP17	70	71.4	20
ISTP18	100	100	100
ISTP19	50	71.4	40
ISTP20	100	100	100
ISTP21	80	57.4	40
ISTP22	40	100	40
ISTP23	30	71.4	60
ISTP24	60	100	40
ISTP25	70	71.4	60
ISTP26	100	100	100
ISTP27	50	57.1	60
ISTP28	70	71.4	40
ISTP29	50	57.1	60

### 3.2 Characteristics of phage ISTP3

**Figure 2:**
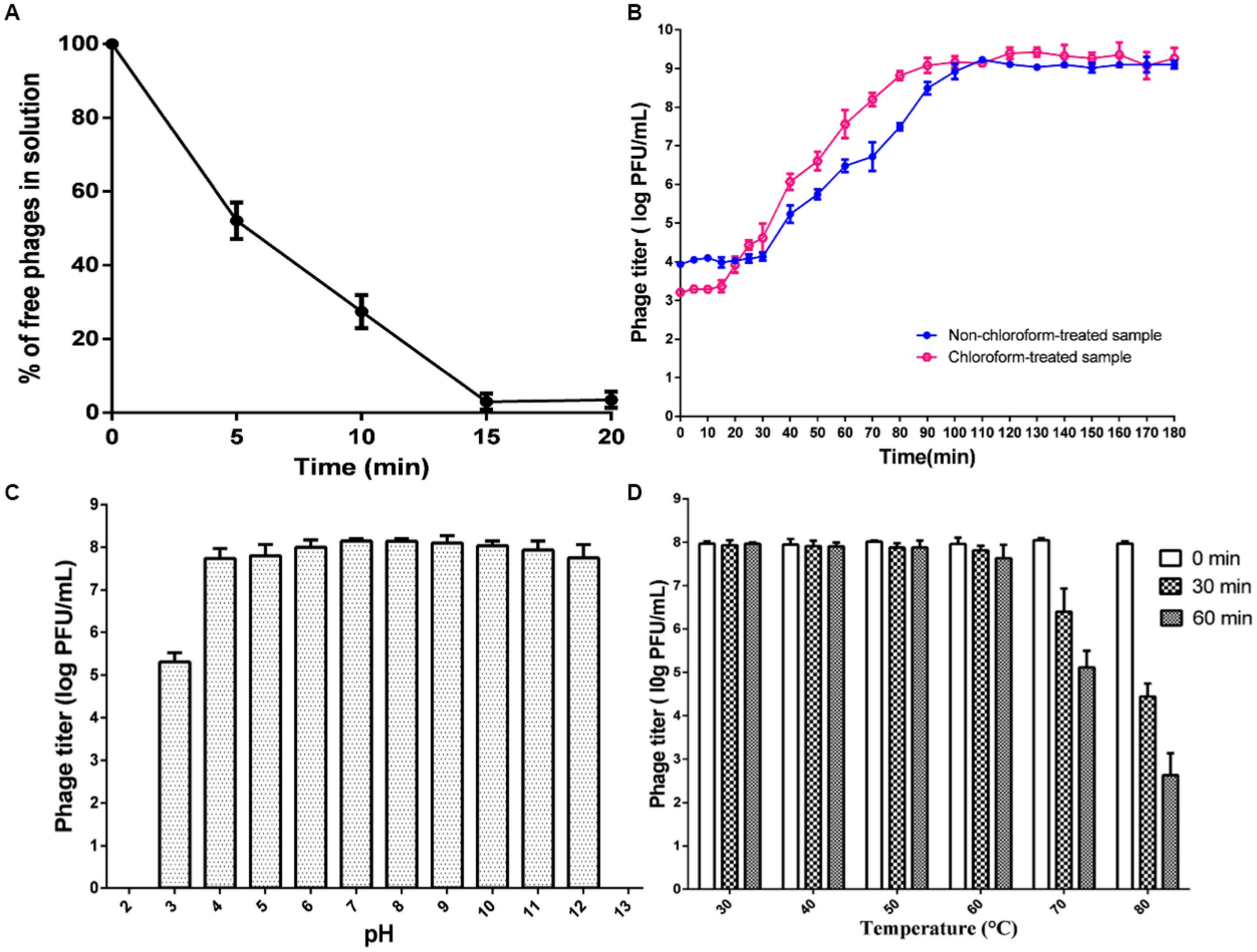
Characteristics of ISTP3. **(A)** Absorption rate. **(B)** One-step growth curve. **(C)** Effect of pH. **(D)** Effect of temperature.

The characteristics of phage ISTP3 are shown in Figure 2. The results of adsorption rate of this phage was show in Figure 2A. About 52% of the phage ISTP3 was adsorbed with host bacteria after 5 min, and accelerated to 97% after 15 min. The one-step growth curve analysis of phage ISTP3 revealed a latent period of around 30 min, an eclipse period of approximately 20 min, and an average burst size of 137 ± 7 PFU (plaque-forming units) per cell (Figure 2B). Using the phage ISTP3 as a biocontrol agent in different food processing environment, the stability and feasibility required confirming during diverse stress conditions including pH and temperature. Stability of pH challenging of the ISTP3 exhibited that it is extremely stable between pH 4 to 12. Nonetheless, the phage titers exhibited a slight decline when the pH was less than 4. However, a reduction was observed at pH 13, with levels dropping to less than 1 log10 PFU/mL (Figure 2C). Interestingly, there was no significant reduce of titer of phage ISTP3 when this phage was kept at 30°C to 60°C for 1 h. The optimum temperature activity of phage was designate between 30°C and 50°C. The phage titers were lost by about 40 and 70% during incubated at 70°C and 80°C, respectively (Figure 2D). The capability of phage ISTP3 for survival in the extensive range of pH and temperatures conditions supports its ability for using in different processing environments and applications as a biocontrol agent for *Salmonella*.

### 3.3 Relative replication efficiency of phage ISTP3

According to Mirzaei et al., single dilution spot tests can yield misleading positive outcomes due to factors such as lysis from outside, presence of bacteriocins in the phage lysate, or the presence of prophages (Islam et al., 2020b). We conducted the EOP test to verify the host range of phage ISTP3 (Table 3). This particular phage demonstrated a significant ability, ranging from 0.5 to less than 1.0, to infect most strains of *S. typhimurium*. However, its effectiveness, as measured by the efficiency of plating (EOP) values, was moderate, ranging from 0.2 to less than 0.5, against certain *S. typhimurium* strains and all *S. enteritidis* strains. The phage demonstrates equal effectiveness against both drug-resistant and non-resistant *Salmonella* strains. The EOP of the ISTP3 phage was examined on seven *Salmonella* strains that are resistant to drugs in our collection. We observed distinct plaques for all seven strains, with relative EOP values varying between 0.2 and 1. The findings indicate that the phage ISTP3 can cause the lysis of various zoonotic *Salmonella* strains.

**Table 3:** Efficiency of plating (EOP) by phage ISTP3 against different *Salmonella* serovars including drug-resistant *Salmonella*.

Bacterial strains	EOP by ISTP3	Drug-resistance *Salmonella*	EOP by ISTP3
*S. enterica* serovar Typhimurium		*S. enterica* serovar Typhimurium	
ATCC 14028	0.9	SA32	Host
ATCC 13311	0.8	SA33	0.6
UK-1	0.8	SA34	0.9
ST8	0.4	SA35	0.7
SGSC 4903	0.6	SA36	0.7
SL 1344	0.3	*S. enterica* serovar Enteritidis	
LT2	0.4	SA29	0.4
*S. enterica* serovar Enteritidis		SA30	0.3
ATCC 13076	0.4		
SJTUF 10978	0.35		
SJTUF 10984	0.3		
LK5-3820	0.45		
SGSC 4901	0.4		

High efficiency, EOP 0.5 to 1.0; moderate efficiency, EOP 0.2 to < 0.5; low efficiency, 0.001 to <0.2; and inefficient, <0.001.

### 3.4 Genome sequencing and bioinformatics analysis

**Figure 3:**
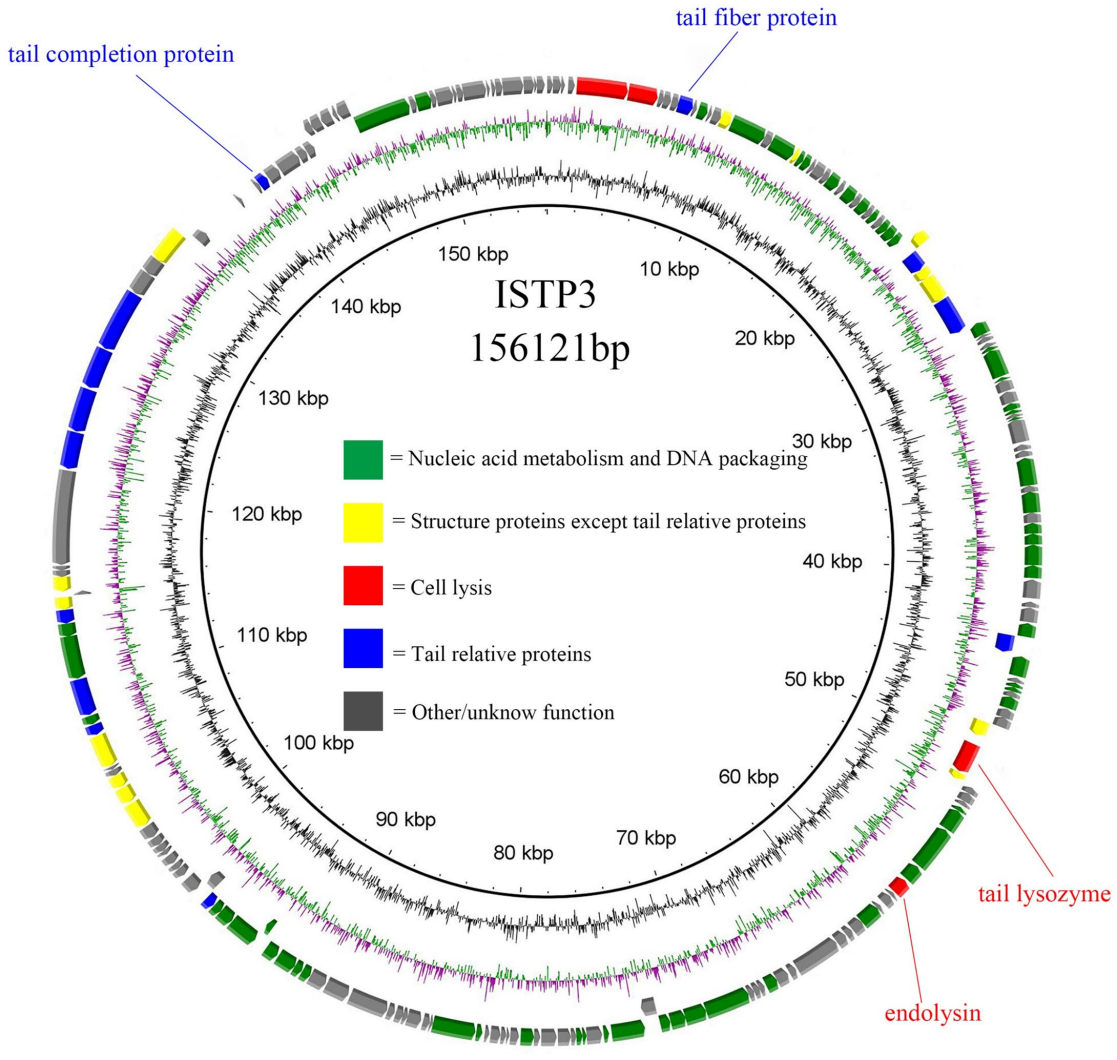
Genome map of phage ISTP3. ORFs set down in the clockwise or the counterclockwise direction. Structural proteins of phage, nucleic acid metabolism, cell lysis, and tail relative protein marked in yellow, green, red, and blue, respectively.

The entire genetic material of phage ISTP3 consists of a 156,121 bp long double-stranded DNA, with a G + C content of 44.78% (Figure 3). The genome sequence of ISTP3 exhibited significant similarity to *Salmonella* phage bering, with a coverage of 90% and an identification rate of 97.28% (Supplementary Table S1). In the ISTP3 genome, a total of 204 open reading frames (ORFs) were found, as indicated in Supplementary Table S2. Out of these, 91 ORFs were determined to be responsible for encoding functional proteins, while the remaining 113 ORFs were found to encode proteins with unidentified functions. Within the group of 91 functional genes, 60 of them were associated with nucleic acid metabolism and DNA packaging, 14 were related to structural proteins other than tails, 4 were cleavage-related genes, and 13 were associated with tail-related proteins, as shown in Figure 3. In the gene annotation of phage ISTP3, we have identified a protein that possesses an Ig-like domain and functions as a tail-related tail fiber protein. This protein has been reported to have associations with phages and has the ability to bind to the human intestinal mucosa, aiding the body in its defense against infections. However, the precise role of the Ig-like protein in the interaction between the phage and the human immune system remains uncertain. The ISTP3 phage does not contain any known virulence or antibiotic resistance genes, indicating that it is a safe phage. Consequently, ISTP3 can be employed in various applications for the control of drug-resistant *Salmonella* in a safe manner.

**Figure 4:**
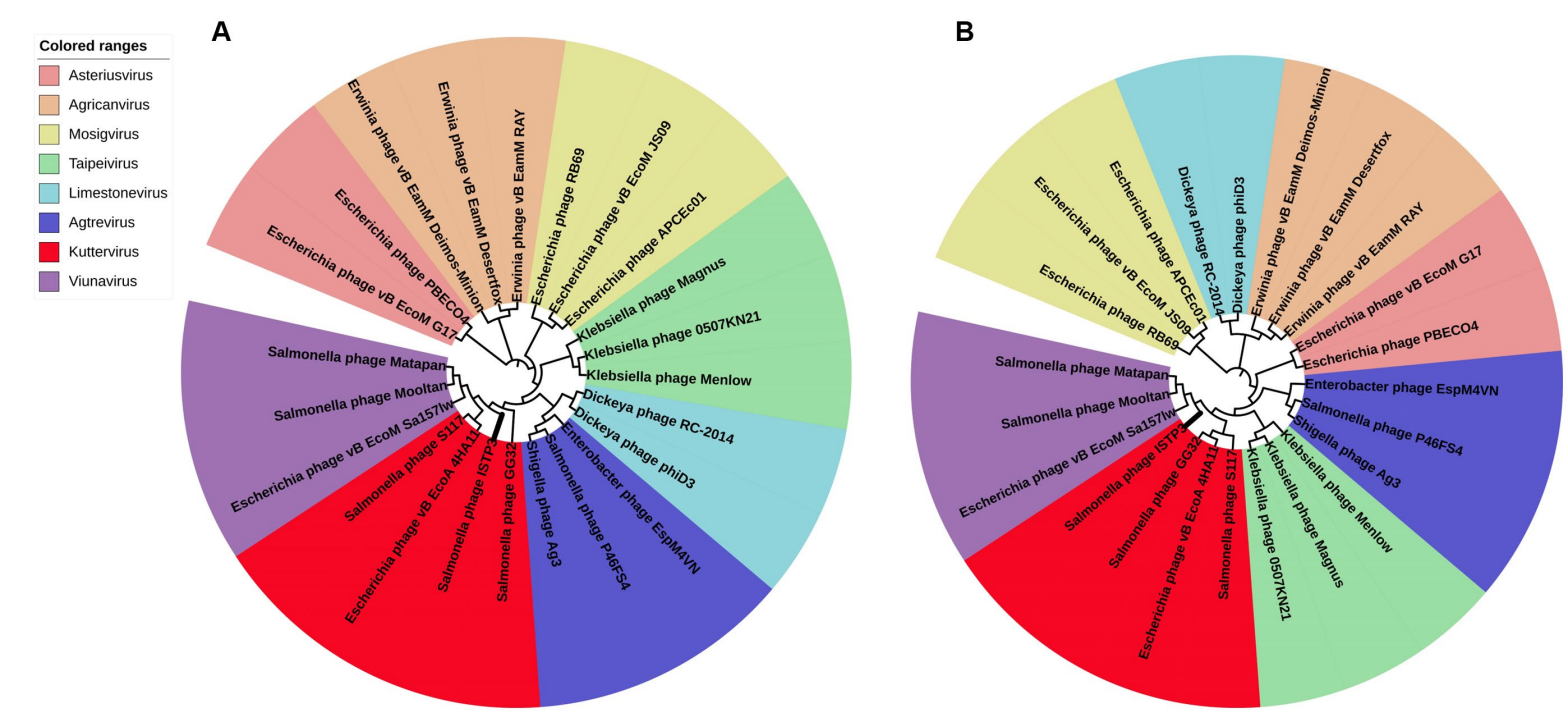
Phylogenetic analysis of phage ISTP3 and related phages based on protein sequence of **(A)** major capsid and **(B)** terminase large subunit.

Given that the structure and fundamental features of the large terminal enzyme subunit tend to be conserved in tail phages, it is expected that phages of the same genus will have clusters of their terminal large subunits. Due to the relative conservation, the major capsid proteins are also very suitable, and there is almost no evidence of horizontal exchange in the head gene region of the phage. The main capsid protein and the terminase large subunit of the phage ISTP3 were compared by BLASTP, and 100 sequences with higher homology were downloaded, and the MEGA7 software was used to select the branches with higher reliability for phylogenetic analysis. ISTP3 forms a same cluster with the main capsid protein and the terminase large subunit of *Salmonella* phage S117, GG32 and *E. coli* phage 4HA1 (Figure 4). According to International Committee on Taxonomy of Viruses (ICTV) classification these four phages belonged to *Ackermannviridae*, which are very closely relationship in cluster.

### 3.5 Evaluating the efficacy of phage ISTP3 to reduce the drug resistant *S. typhimurium* in foods

**Figure 5:**
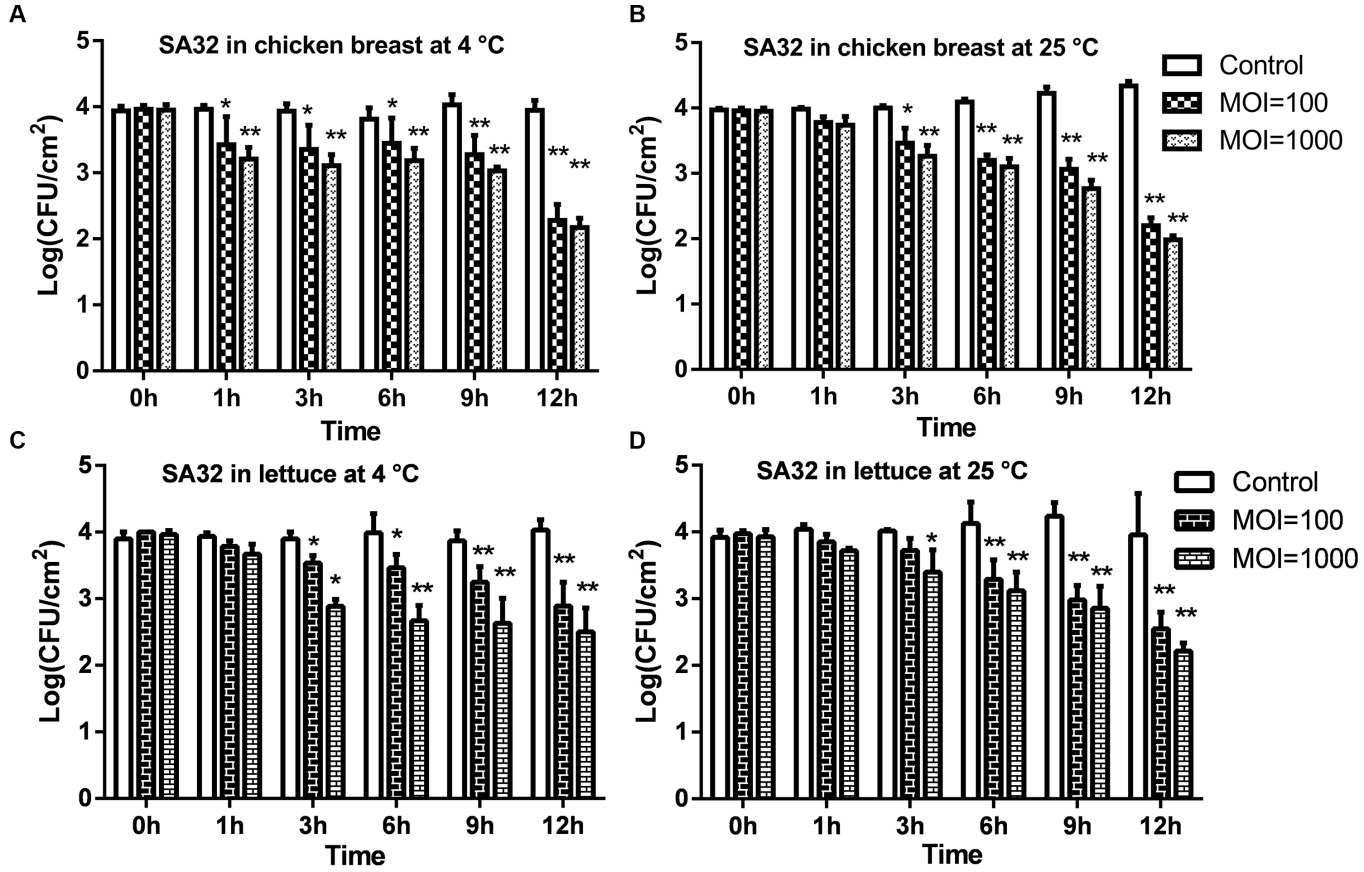
The application of phage ISTP3 to reduce the *S. typhimurium* on food matrices. The efficacy of phage was tested chicken breast and lettuce with bacteria using phage treated. Viable bacteria were counted at 0, 1, 3, 6, 9, 12 h. **(A)** Viable *S. typhimurium* on the chicken breast at 4°C. **(B)** Viable *S. typhimurium* on the chicken breast at 25°C. **(C)** Viable *S. typhimurium* on lettuce at 4°C. **(D)** Viable *S. typhimurium* on lettuce at 25°C. Control samples; without phage treated; MOI = 100, samples were treated with 6 log PFU of ISTP3 and 4 log CFU bacteria; MOI = 1,000, samples were treated with 7 log PFU of ISTP3 and 4 log CFU bacteria. *p < 0.05 and **p < 0.01.

It has been found that the phage ISTP3 could lyse the drug-resistant *Salmonella* during the evaluation of host spectrum, so foods have been contaminated with drug-resistant *S. typhimurium* in the application experiments. The experimental findings regarding chicken breast are represented in Figures 5A,B. When compared to the control group, a notable decrease of 1.6 log CFU/cm^2^ and 1.8 log CFU/cm^2^ in viable drug-resistant *S. typhimurium* was observed after 12 h at 4°C, with MOIs of 100 and 1,000, respectively (Figure 5A). At a temperature of 25°C, the population of viable *S. typhimurium* decreased by 2.1 log CFU/cm^2^ and 2.4 log CFU/cm^2^ when exposed to MOIs of 100 and 1,000 respectively, following a 12 h incubation period (as shown in Figure 5B).

The antibacterial effectiveness of the ISTP3 phage in reducing drug-resistant *S. typhimurium* was investigated through artificial contamination of bacteria on the surface of lettuce. Phage ISTP3 demonstrated its effectiveness in reducing bacterial counts on lettuce samples when incubated at 4°C for 12 h. At MOIs of 100 and 1,000, viable cell counts decreased by at least 1.1 log CFU/cm^2^ and 1.5 log CFU/cm^2^ respectively, as shown in Figure 5C. The application of a MOI of 100 and 1,000 resulted in a reduction of viable bacteria at 25°C after 12 h. Specifically, there was a decrease of 1.4 log 10 CFU/cm^2^ and 1.7 log CFU/cm^2^, respectively, at the end of the 12 h incubation period (as shown in Figure 5D).

## 4 Discussion

*Salmonella* poses a significant challenge in terms of outbreaks of zoonotic pathogens within the food industry, impacting safety measures. While phage usage offers the key advantage of specificity towards its target bacteria, allowing non-target bacterial populations to remain undisturbed, phages are still capable of lysing the majority of strains belonging to a particular bacterial species (Almeida et al., 2009). Phage has been intended as a promising green technology for biocontrol of different foodborne pathogenic bacteria. We isolated 29 phages capable of lysing various strains of *Salmonella*, and during screening for effectiveness against different *Salmonella* serovars, phage ISTP3 demonstrated a broad host range activity by successfully lysing all tested *Salmonella* serovars and achieving complete clearing. Furthermore, bacteriophages exhibiting a broad host range, capable of lysing various serovars of pathogenic bacteria, including diverse strains, prove highly effective in the context of food and food processing environments. In our study, we assessed the host range of phage ISTP3 against various *Salmonella* serovars, including drug-resistant strains. The results demonstrated that phage ISTP3 was able to lyse all 31 tested *Salmonella* strains, including 10 drug-resistant strains. These findings provide evidence of the potential of phage therapy in controlling drug-resistant zoonotic *Salmonella*, offering a promising solution for combating antibiotic-resistant strains. The significance of having a broad host range, which enables the phage to interact and replicate efficiently with various bacterial hosts, has been well established. This criterion is considered crucial in determining the suitability of a phage as a leading candidate in terms of its dynamics with host bacteria (Li et al., 2021). The categorical differentiation between various stages of the broad host range is not clearly explained. Recent studies by Dekel-Bird et al. (2015), Kauffman et al. (2018) focusing on newly isolated marine broad-host-range phage, as well as the examination of extreme environments through community-wide single-cell metagenomics by Munson-McGee et al. (2018), have provided evidence of the widespread presence of broad host range phages in natural environments, as supported by Brum et al. (2016), Paez-Espino et al. (2016). Host range analysis is a crucial factor when choosing lytic phages for potential utilization in the biocontrol purposes. Additionally, the EOP results indicate that phage ISTP3 demonstrated the capability to lyse not only its host but also all the tested *Salmonella* strains. In our research, we demonstrated that the tailed phage ISTP3 displayed a broad range of hosts, which may be attributed to the abundance or superior quality of *Salmonella* strains, including those resistant to drugs, coexisting with phage ISTP3 in various environments.

In this study, we selected the most virulent phage with the broadest host range, which is capable of lysing diverse zoonotic *Salmonella* strains within our collection, for potential biocontrol applications. Upon TEM examination, it was determined that phage ISTP3 belongs to the *Caudovirales* order and *Ackermannviridae* family. Other studies (Hooton et al., 2011; Yele et al., 2012; Sylwia et al., 2014; Novacek et al., 2016; Sunderland et al., 2017; Islam et al., 2019a) have suggested that phages within this family possess the potential to be used as biocontrol candidates against *Salmonella*. The process of phage adsorption, which involves the attachment of phage particles to host cells, is influenced by multiple factors such as the characteristics of the receptor, its position within the cell wall, ease of access, arrangement in space, as well as the abundance and concentration at different sites. Phage ISTP3 exhibits a shorter latent period and a larger burst during its one-step growth curve. These characteristics make it more advantageous for biocontrol purposes as it can effectively and quickly propagate, leading to the rapid elimination of the targeted bacterial population (Duc et al., 2020). The capacity of phages to serve as biocontrol agents across diverse environmental conditions is enhanced by their stability. In this study, the *Salmonella* phage ISTP3, isolated for evaluation, underwent incubation at various pH levels and temperatures to assess its ability to maintain infectivity. Phage ISTP3 demonstrated tolerated within a pH range of 4 to 12 for a duration of 60 min, as its viral titers remained largely unaffected. Previous research indicates that many phages have an optimal pH range of 6 to 8, although certain phages like T2 phage experience a 50% decline in infectivity between pH levels of 5 and 9 (Jończyk et al., 2011). On the other hand, the present study revealed that phage ISTP3 maintained its titer without any notable decrease when exposed to temperatures ranging from 30°C to 60°C for a period of 60 min.

Further genomic sequencing and bioinformatics analysis of phage ISTP3 confirmed that it is a member of the *Ackermannviridae* family, classified as a double-stranded DNA virus. However, upon examination, it was observed that the ISTP3 genome lacks any recognized lysogenic genes, which are vital criteria for a lytic phage to effectively target and eliminate pathogens. Additionally, no ORFs were detected that were linked to pathogenicity or the production of toxins, rendering the phage unsuitable for therapeutic purposes. These findings substantiate the potential of the ISTP3 phage for applications in bio-control and ensuring safety (Alves et al., 2020).

In the realm of managing *Enterobacteriaceae*, particularly *S. enterica*, which stands out as the most frequently encountered zoonotic microorganism, phages are commonly employed as a means of control. Several studies have documented the identification and usage of lytic phages to combat prevalent foodborne pathogens, such as *E. coli* O157:H7 (Hudson et al., 2013), *Salmonella* (Guenther et al., 2012), *Listeria monocytogenes* (Bigot et al., 2011), *Campylobacter* (Loc Carrillo et al., 2005), *Enterobacter sakazaki* (Zuber et al., 2008), and *Staphylococcus aureus* (García et al., 2009). To our knowledge, there are very few studies to evaluate efficacy of phage to control drug-resistant *Salmonella* in foods. In this study we used phage ISTP3 for efficiently reducing the drug resistant *S. typhimurium* SA32 in food matrices at 4°C or 25°C. When applied to chicken breast samples, phage ISTP3 exhibited a reduction of *Salmonella* by over 1.5 log at 4°C and over 2 log at 25°C within a 12 h incubation period. Diverse studies have provided evidence of employing individual phages, such as LPST153, ZCSE2, ST-W77, BSP101, SI1, and S144, specifically to mitigate *Salmonella* contamination in food items (Fong et al., 2017; Bai et al., 2019; Gambino et al., 2020; Mohamed et al., 2020; Phothaworn et al., 2020; Islam et al., 2020a). Typically, increasing the MOI (multiplicity of infection) on food samples leads to a greater decrease in pathogens. Theoretically, using high phage titers would enhance the chances of phages interacting with the target bacteria. Numerous studies have shown the effectiveness of applying phages to meat under low-temperature conditions (Carter et al., 2012; Hudson et al., 2013). The successful inactivation of *Salmonella* at low temperatures and with high MOI values may be attributed to the mechanism known as lysis from without (LO), as proposed in previous research (David Tomat et al., 2018).

Additionally, the application of ISTP3 phage treatment has demonstrated a significant reduction in drug-resistant *S. enterica* on lettuce. After 12 h of incubation at both 4°C and 25°C, viable cell counts decreased by approximately 1 to 1.8 log. The findings of the current study align with previous research evaluating the effectiveness of ECP-100 phage on fresh-cut lettuce. In a study conducted by Li et al. (2021), it was observed that the application of phage ECP-100 resulted in a reduction of bacterial populations by approximately 1.82 log CFU/cm^2^. Complete eradication of bacteria through phage application may not be achieved. However, the significant reduction in viable bacterial counts following phage therapy suggests that it may lower the bacterial load below the infectious dose for individuals.

## 5 Conclusion

In this study, the phage ISTP3 was isolated and it is fully active at 60°C for 1 h which is defined as thermostable. This particular phage exhibited a wide host range and demonstrated the capacity to effectively lyse drug-resistant strains of *S. enterica*. ISTP3 did not contain any virulent genes, lysogenic, or drug-resistance which are make it safe for application on food matrices. Additionally, the phage ISTP3 achieved reductions of drug-resistant *S. enterica* on chicken breast and lettuce, making it an efficient biocontrol agent for food and food processing chain which may offer one of the safe and environmentally friendly approach. The phage treatment is optimizing novel strategies for the control of drug-resistant *Salmonella* contamination on chicken and fresh produce and it could reduce the economic loss and burden of health associated with diverse outbreaks.

## Data availability statement

The original contributions presented in the study are included in the article/Supplementary material, further inquiries can be directed to the corresponding authors.

## Author contributions

MSI: Conceptualization, Data curation, Formal analysis, Investigation, Methodology, Project administration, Resources, Software, Validation, Visualization, Writing – original draft, Writing – review & editing. IN: Conceptualization, Data curation, Formal analysis, Methodology, Software, Writing – original draft. FP: Conceptualization, Funding acquisition, Investigation, Project administration, Resources, Supervision, Validation, Writing – review & editing. XW: Conceptualization, Funding acquisition, Investigation, Project administration, Resources, Supervision, Validation, Writing – review & editing.

## Footnotes

^1^ http://www.gatan.com/products/tem-analysis/gatan-microscopy-suite-software

^2^ https://cge.cbs.dtu.dk/services/KmerFinder/

^3^ https://www.mgc.ac.cn/VFs/main.htm
