# Supplementary material for: Isolation and characterization of phage ISTP3 for bio-control application against drug-resistant Salmonella
Source: Front Microbiol. 2023 Nov 22;14:1260181. doi: 10.3389/fmicb.2023.1260181 (PMC10702751; doi:10.3389/fmicb.2023.1260181)
Supplement: Supplementary file 1 [file Data_Sheet_1.docx]

**Isolation and characterization of phage ISTP3 for bio-control application against drug-registrant *Salmonella***

Md. Sharifull Islam^1,2†^, Ishatur Nime^1†^, Fan Pan^2,^*, and Xiaohong Wang^1,^*

1. Key Laboratory of Environment Correlative Dietology, College of Food Science and Technology, Huazhong Agricultural University, Wuhan, Hubei, China
2. Center for Cancer Immunology, Institute of Biomedicine and Biotechnology, Shenzhen Institute of Advanced Technology, Chinese Academy of Sciences, Shenzhen 518055, China

† The authors contributed equally to this work.

***** Correspondence: wxh@mail.hzau.edu.cn (wxh); fan.pan@siat.ac.cn (FP)

TABLE S1 The ISTP3 phage genome sequences compared BLASTN analysis at NCBI

| Phage | BLASTN  Score | | Total Score | Query  Cover | E  value | Per.  Identify | Accession |
| --- | --- | --- | --- | --- | --- | --- | --- |
| Salmonella phage S117 | 55105 | 2.360e+05 | | 90% | 0.0 | 97.09% | [MH370370.1](https://www.ncbi.nlm.nih.gov/nucleotide/MH370370.1?report=genbank&log$=nucltop&blast_rank=1&RID=MW8WHWDH014) |
| Salmonella phage bering | 43535 | | 2.193e+05 | 90% | 0.0 | 97.28% | [NC_049502.1](https://www.ncbi.nlm.nih.gov/nucleotide/NC_049502.1?report=genbank&log$=nucltop&blast_rank=2&RID=MXY4SJJH016) |
| Escherichia phage vB_EcoA_4HA11 | 42968 | | 2.098e+05 | 85% | 0.0 | 96.35% | [MN445184.1](https://www.ncbi.nlm.nih.gov/nucleotide/MN445184.1?report=genbank&log$=nucltop&blast_rank=3&RID=MXY4SJJH016) |
| Salmonella phage STP07 | 39944 | | 2.368e+05 | 89% | 0.0 | 97.23% | [KY000003.1](https://www.ncbi.nlm.nih.gov/nucleotide/KY000003.1?report=genbank&log$=nucltop&blast_rank=7&RID=MXY4SJJH016) |
| Salmonella phage GG32 | 39938 | | 2.334e+05 | 89% | 0.0 | 97.23% | [KX245012.1](https://www.ncbi.nlm.nih.gov/nucleotide/KX245012.1?report=genbank&log$=nucltop&blast_rank=8&RID=MXY4SJJH016) |
| Salmonella phage Mooltan | 37172 | | 1.946e+05 | 94% | 0.0 | 92.82% | [MH688040.1](https://www.ncbi.nlm.nih.gov/nucleotide/MH688040.1?report=genbank&log$=nucltop&blast_rank=18&RID=MXY4SJJH016) |
| Salmonella phage S8 | 32576 | | 2.381e+05 | 91% | 0.0 | 97.38% | [KY630163.1](https://www.ncbi.nlm.nih.gov/nucleotide/KY630163.1?report=genbank&log$=nucltop&blast_rank=20&RID=MXY4SJJH016) |
| Salmonella phage SE14 | 31990 | | 2.310e+05 | 90% | 0.0 | 95.98% | [MK972690.1](https://www.ncbi.nlm.nih.gov/nucleotide/MK972690.1?report=genbank&log$=nucltop&blast_rank=24&RID=MXY4SJJH016) |
| Escherichia phage vB_EcoM_3HA11 | 30869 | | 1.972e+05 | 91% | 0.0 | 91.35% | [MN342150.1](https://www.ncbi.nlm.nih.gov/nucleotide/MN342150.1?report=genbank&log$=nucltop&blast_rank=31&RID=MXY4SJJH016) |
| Salmonella phage SenASZ3 | 30710 | | 2.283e+05 | 90% | 0.0 | 95.63% | [NC_049439.1](https://www.ncbi.nlm.nih.gov/nucleotide/NC_049439.1?report=genbank&log$=nucltop&blast_rank=33&RID=MXY4SJJH016) |
| Salmonella phage Mutine | 28068 | | 2.142e+05 | 89% | 0.0 | 95.71% | [MG428992.1](https://www.ncbi.nlm.nih.gov/nucleotide/MG428992.1?report=genbank&log$=nucltop&blast_rank=36&RID=MXY4SJJH016) |
| Salmonella phage PS5 | 27850 | | 1.995e+05 | 94% | 0.0 | 92.98% | [MH940212.1](https://www.ncbi.nlm.nih.gov/nucleotide/MH940212.1?report=genbank&log$=nucltop&blast_rank=39&RID=MXY4SJJH016) |
| Escherichia phage EP75 | 27436 | | 2.078e+05 | 89% | 0.0 | 92.28% | [MG748547.1](https://www.ncbi.nlm.nih.gov/nucleotide/MG748547.1?report=genbank&log$=nucltop&blast_rank=42&RID=MXY4SJJH016) |
| Salmonella phage S115 | 26482 | | 2.203e+05 | 89% | 0.0 | 94.09% | [MH370368.1](https://www.ncbi.nlm.nih.gov/nucleotide/MH370368.1?report=genbank&log$=nucltop&blast_rank=45&RID=MXY4SJJH016) |
| Salmonella phage STML-13-1 | 24496 | | 1.894e+05 | 90% | 0.0 | 89.14% | [NC_042061.1](https://www.ncbi.nlm.nih.gov/nucleotide/NC_042061.1?report=genbank&log$=nucltop&blast_rank=49&RID=MXY4SJJH016) |
| Salmonella phage vB_SalM_SJ2 | 18862 | | 1.825e+05 | 86% | 0.0 | 93.30% | [KJ174317.1](https://www.ncbi.nlm.nih.gov/nucleotide/KJ174317.1?report=genbank&log$=nucltop&blast_rank=58&RID=MXY4SJJH016) |
| Escherichia phage vB_EcoM_KWBSE43-6 | 7812 | | 45936 | 51% | 0.0 | 80.20% | [NC_048186.1](https://www.ncbi.nlm.nih.gov/nucleotide/NC_048186.1?report=genbank&log$=nucltop&blast_rank=65&RID=MXY4SJJH016) |
| Klebsiella phage May | 7805 | | 43699 | 48% | 0.0 | 80.16% | [NC_047900.1](https://www.ncbi.nlm.nih.gov/nucleotide/NC_047900.1?report=genbank&log$=nucltop&blast_rank=66&RID=MXY4SJJH016) |
| Shigella phage phiSboM-AG3 | 5384 | | 64428 | 62% | 0.0 | 82.66% | [NC_013693.1](https://www.ncbi.nlm.nih.gov/nucleotide/NC_013693.1?report=genbank&log$=nucltop&blast_rank=75&RID=MXY4SJJH016) |
| Klebsiella phage Magnus | 4588 | | 42155 | 46% | 0.0 | 79.97% | [MN045230.1](https://www.ncbi.nlm.nih.gov/nucleotide/MN045230.1?report=genbank&log$=nucltop&blast_rank=80&RID=MXY4SJJH016) |
| Dickeya phage RC-2014 | 2575 | | 46893 | 49% | 0.0 | 76.22% | [KJ716335.1](https://www.ncbi.nlm.nih.gov/nucleotide/KJ716335.1?report=genbank&log$=nucltop&blast_rank=87&RID=MXY4SJJH016) |
| Dickeya phage phiD3 | 2196 | | 46610 | 48% | 0.0 | 76.39% | [KM209228.1](https://www.ncbi.nlm.nih.gov/nucleotide/KM209228.1?report=genbank&log$=nucltop&blast_rank=94&RID=MXY4SJJH016) |

| TABLE S2 Open reading frames (ORFs) of ISTP3 | | | | | | | |  |
| --- | --- | --- | --- | --- | --- | --- | --- | --- |
|  |  |  |  |  |  |  |  |  |
| ORF | Functions | Gene Positions | Putative conserved domains | | | | |  |
|  |  |  | Name | Accession | Description | Interval | E-value |  |
| 1 | [hypothetical protein](https://blast.ncbi.nlm.nih.gov/Blast.cgi#alnHdr_YP_009140344) | \|+\|1\|309 |  |  |  |  |  |  |
| 2 | [hypothetical protein](https://blast.ncbi.nlm.nih.gov/Blast.cgi#alnHdr_YP_009879620) | \|+\|306\|752 |  |  |  |  |  |  |
| 3 | [hypothetical protein](https://blast.ncbi.nlm.nih.gov/Blast.cgi#alnHdr_YP_009140342) | \|+\|745\|951 |  |  |  |  |  |  |
| 4 | [hypothetical protein](https://blast.ncbi.nlm.nih.gov/Blast.cgi#alnHdr_YP_009030414) | \|+\|1121\|1474 |  |  |  |  |  |  |
| 5 | [rIIA lysis inhibitor](https://blast.ncbi.nlm.nih.gov/Blast.cgi#alnHdr_YP_009283819) | \|+\|1582\|4317 |  |  |  |  |  |  |
| 6 | [rIIB lysis inhibitor](https://blast.ncbi.nlm.nih.gov/Blast.cgi#alnHdr_QEI24152) | \|+\|4350\|5933 | HTH super family | [cl21459](https://www.ncbi.nlm.nih.gov/Structure/cdd/cddsrv.cgi?ascbin=8&maxaln=10&seltype=2&uid=cl21459) | Helix-turn-helix domains | 48-86 | 2.94E-05 |  |
| 7 | [hypothetical protein](https://blast.ncbi.nlm.nih.gov/Blast.cgi#alnHdr_YP_008771026) | \|+\|5987\|6286 |  |  |  |  |  |  |
| 8 | [hypothetical protein](https://blast.ncbi.nlm.nih.gov/Blast.cgi#alnHdr_YP_009030409) | \|+\|6261\|6674 |  |  |  |  |  |  |
| 9 | [hypothetical protein](https://blast.ncbi.nlm.nih.gov/Blast.cgi#alnHdr_ANT44611) | \|+\|6707\|7099 | PHA02335 super family | [cl10423](https://www.ncbi.nlm.nih.gov/Structure/cdd/cddsrv.cgi?ascbin=8&maxaln=10&seltype=2&uid=cl10423) | hypothetical protein | 4-115 | 1.01E-08 |  |
| 10 | [tail fiber protein](https://blast.ncbi.nlm.nih.gov/Blast.cgi#alnHdr_ARB06394) | \|+\|7078\|7887 | YjdB super family  Ig_3 | [cl35007 pfam13927](https://www.ncbi.nlm.nih.gov/Structure/cdd/cddsrv.cgi?ascbin=8&maxaln=10&seltype=2&uid=cl35007) | Uncharacterized conserved protein YjdB, contains Ig-like domain [General function prediction only] Immunoglobulin domain; This family contains immunoglobulin-like domains. | 79-199 179-252 | 0.0000004 0.000281 |  |
|  |  |  |  |  |  |  |  |  |
| 11 | [hypothetical protein](https://blast.ncbi.nlm.nih.gov/Blast.cgi#alnHdr_YP_009140334) | \|+\|7891\|8112 |  |  |  |  |  |  |
| 12 | [HU family DNA-binding protein](https://blast.ncbi.nlm.nih.gov/Blast.cgi#alnHdr_EDW4918034) | \|+\|8200\|8709 | HU_IHF super family | [cl00257](https://www.ncbi.nlm.nih.gov/Structure/cdd/cddsrv.cgi?ascbin=8&maxaln=10&seltype=2&uid=cl00257) | DNA sequence specific (IHF) and non-specific (HU) domains | 80-108 | 7.04E-03 |  |
| 13 | [hypothetical protein](https://blast.ncbi.nlm.nih.gov/Blast.cgi#alnHdr_YP_009140332) | \|+\|8757\|8948 |  |  |  |  |  |  |
| 14 | [hypothetical protein](https://blast.ncbi.nlm.nih.gov/Blast.cgi#alnHdr_AXC40722) | \|+\|8990\|9475 |  |  |  |  |  |  |
| 15 | [viral tegument-like protein](https://blast.ncbi.nlm.nih.gov/Blast.cgi#alnHdr_YP_009887688) | \|+\|9472\|10050 |  |  |  |  |  |  |
| 16 | [DNA topoisomerase II large subunit](https://blast.ncbi.nlm.nih.gov/Blast.cgi#alnHdr_YP_009887687) | \|+\|10100\|12013 | PTZ00108 super family | [cl36510](https://www.ncbi.nlm.nih.gov/Structure/cdd/cddsrv.cgi?ascbin=8&maxaln=10&seltype=2&uid=cl36510) | DNA topoisomerase 2-like protein; Provisional | 1-625 | 1.60E-142 |  |
| 17 | [hypothetical protein](https://blast.ncbi.nlm.nih.gov/Blast.cgi#alnHdr_YP_008771018) | \|+\|12015\|12464 | MoCo_carrier super family | [cl15040](https://www.ncbi.nlm.nih.gov/Structure/cdd/cddsrv.cgi?ascbin=8&maxaln=10&seltype=2&uid=cl15040) | Putative molybdenum carrier | 99-146 | 2.57E-03 |  |
| 18 | [DNA topoisomerase medium subunit](https://blast.ncbi.nlm.nih.gov/Blast.cgi#alnHdr_YP_009881923) | \|+\|12461\|13786 | 52 super family | [cl29577](https://www.ncbi.nlm.nih.gov/Structure/cdd/cddsrv.cgi?ascbin=8&maxaln=10&seltype=2&uid=cl29577) | DNA topisomerase II medium subunit; Provisional | 1-437 | 0.00E+00 |  |
| 19 | [membrane protein](https://blast.ncbi.nlm.nih.gov/Blast.cgi#alnHdr_YP_009030399) | \|+\|13829\|14107 |  |  |  |  |  |  |
| 20 | [anaerobic dehydrogenase](https://blast.ncbi.nlm.nih.gov/Blast.cgi#alnHdr_ECW1086640) | \|+\|14109\|14420 |  |  |  |  |  |  |
| 21 | [tRNA processing enzyme/C-N hydrolase superfamily protein](https://blast.ncbi.nlm.nih.gov/Blast.cgi#alnHdr_AXF41638) | \|+\|14424\|14837 |  |  |  |  |  |  |
| 22 | [hypothetical protein](https://blast.ncbi.nlm.nih.gov/Blast.cgi#alnHdr_YP_009293421) | \|+\|14890\|15126 |  |  |  |  |  |  |
| 23 | [hypothetical protein](https://blast.ncbi.nlm.nih.gov/Blast.cgi#alnHdr_YP_007007995) | \|+\|15123\|15728 | Macro super family | [cl00019](https://www.ncbi.nlm.nih.gov/Structure/cdd/cddsrv.cgi?ascbin=8&maxaln=10&seltype=2&uid=cl00019) | Macro domain | 25-145 | 5.13E-27 |  |
| 24 | [hypothetical protein](https://blast.ncbi.nlm.nih.gov/Blast.cgi#alnHdr_EDL0982001) | \|+\|15725\|16069 |  |  |  |  |  |  |
| 25 | [exonuclease](https://blast.ncbi.nlm.nih.gov/Blast.cgi#alnHdr_AXF41633) | \|+\|16062\|16694 | DnaQ_like_exo super family | [cl10012](https://www.ncbi.nlm.nih.gov/Structure/cdd/cddsrv.cgi?ascbin=8&maxaln=10&seltype=2&uid=cl10012) | DnaQ-like (or DEDD) 3'-5' exonuclease domain superfamily | 9-206 | 7.11E-09 |  |
| 26 | [hypothetical protein](https://blast.ncbi.nlm.nih.gov/Blast.cgi#alnHdr_YP_009880589) | \|+\|16772\|17062 |  |  |  |  |  |  |
| 27 | [alpha hydrolase/argininosuccinate synthase](https://blast.ncbi.nlm.nih.gov/Blast.cgi#alnHdr_ASZ78955) | \|+\|17160\|17822 | AANH_like super family | [cl00292](https://www.ncbi.nlm.nih.gov/Structure/cdd/cddsrv.cgi?ascbin=8&maxaln=10&seltype=2&uid=cl00292) | Adenine nucleotide alpha hydrolases superfamily including N type ATP PPase | 27-58 | 1.01E-03 |  |
| 28 | [hypothetical protein](https://blast.ncbi.nlm.nih.gov/Blast.cgi#alnHdr_YP_007002665) | \|+\|17825\|18166 |  |  |  |  |  |  |
| 29 | [metallophosphoesterase](https://blast.ncbi.nlm.nih.gov/Blast.cgi#alnHdr_WP_021342604) | \|+\|18166\|18726 | MPP_superfamily super family | [cl13995](https://www.ncbi.nlm.nih.gov/Structure/cdd/cddsrv.cgi?ascbin=8&maxaln=10&seltype=2&uid=cl13995) | metallophosphatase superfamily | 9-177 | 1.36E-11 |  |
| 30 | [hypothetical protein](https://blast.ncbi.nlm.nih.gov/Blast.cgi#alnHdr_AXF41628) | \|+\|18723\|19055 |  |  |  |  |  |  |
| 31 | [propeller repeat protein](https://blast.ncbi.nlm.nih.gov/Blast.cgi#alnHdr_WP_147647156) | \|+\|19052\|19402 |  |  |  |  |  |  |
| 32 | [hypothetical protein](https://blast.ncbi.nlm.nih.gov/Blast.cgi#alnHdr_QKE54676) | \|+\|19466\|19807 |  |  |  |  |  |  |
| 33 | [TPA: aldehyde dehydrogenase](https://blast.ncbi.nlm.nih.gov/Blast.cgi#alnHdr_HBF78390) | \|+\|19804\|20211 |  |  |  |  |  |  |
| 34 | [deoxycytidylate deaminase](https://blast.ncbi.nlm.nih.gov/Blast.cgi#alnHdr_YP_009879813) | \|+\|20204\|20722 | cytidine_deaminase-like super family | [cl00269](https://www.ncbi.nlm.nih.gov/Structure/cdd/cddsrv.cgi?ascbin=8&maxaln=10&seltype=2&uid=cl00269) | Cytidine and deoxycytidylate deaminase zinc-binding region. | 6-126 | 2.09E-22 |  |
| 35 | [hypothetical protein/TPA: aldehyde dehydrogenase](https://blast.ncbi.nlm.nih.gov/Blast.cgi#alnHdr_QKE54677) | \|+\|20724\|21131 | bPH_2 | [pfam03703](https://www.ncbi.nlm.nih.gov/Structure/cdd/cddsrv.cgi?ascbin=8&maxaln=10&seltype=2&uid=pfam03703) | Bacterial PH domain; | 56-113 | 1.48E-09 |  |
| 36 | [head completion protein](https://blast.ncbi.nlm.nih.gov/Blast.cgi#alnHdr_YP_009220972) | \|-\|21342\|21962 | Tn7_Tnp_TnsA_N super family | [cl21695](https://www.ncbi.nlm.nih.gov/Structure/cdd/cddsrv.cgi?ascbin=8&maxaln=10&seltype=2&uid=cl21695) | TnsA endonuclease N terminal | 3-163 | 1.86E-66 |  |
| 37 | [baseplate tail tube cap](https://blast.ncbi.nlm.nih.gov/Blast.cgi#alnHdr_YP_009879808) | \|+\|22016\|22984 | BTB_POZ super family | [cl38908](https://www.ncbi.nlm.nih.gov/Structure/cdd/cddsrv.cgi?ascbin=8&maxaln=10&seltype=2&uid=cl38908) | BTB (Broad-Complex | 240-320 | 8.15E-03 |  |
| 38 | [baseplate wedge subunit](https://blast.ncbi.nlm.nih.gov/Blast.cgi#alnHdr_YP_007008011) | \|+\|22997\|23554 | Phage_gp53 super family | [cl26475](https://www.ncbi.nlm.nih.gov/Structure/cdd/cddsrv.cgi?ascbin=8&maxaln=10&seltype=2&uid=cl26475) | Base plate wedge protein 53 | 56-185 | 6.51E-13 |  |
| 39 | [hypothetical protein/baseplate hub component](https://blast.ncbi.nlm.nih.gov/Blast.cgi#alnHdr_ATW62030) | \|+\|23551\|24939 |  |  |  |  |  |  |
| 40 | [tail length tape measure protein](https://blast.ncbi.nlm.nih.gov/Blast.cgi#alnHdr_YP_009798830) | \|+\|24950\|26890 |  |  |  |  |  |  |
| 41 | [DNA helicase loader](https://blast.ncbi.nlm.nih.gov/Blast.cgi#alnHdr_YP_009876076) | \|-\|26891\|27553 | 59 super family | [cl33685](https://www.ncbi.nlm.nih.gov/Structure/cdd/cddsrv.cgi?ascbin=8&maxaln=10&seltype=2&uid=cl33685) | 59 protein; Provisional | 13-194 | 6.23E-10 |  |
| 42 | [hypothetical protein](https://blast.ncbi.nlm.nih.gov/Blast.cgi#alnHdr_YP_009283854) | \|-\|27543\|27707 |  |  |  |  |  |  |
| 43 | [hypothetical protein](https://blast.ncbi.nlm.nih.gov/Blast.cgi#alnHdr_YP_009879802) | \|-\|27697\|27960 |  |  |  |  |  |  |
| 44 | [hypothetical protein](https://blast.ncbi.nlm.nih.gov/Blast.cgi#alnHdr_YP_009888459) | \|-\|28043\|28258 |  |  |  |  |  |  |
| 45 | [transcriptional regulator](https://blast.ncbi.nlm.nih.gov/Blast.cgi#alnHdr_YP_009888458) | \|-\|28255\|28464 | COG3398 super family | [cl28563](https://www.ncbi.nlm.nih.gov/Structure/cdd/cddsrv.cgi?ascbin=8&maxaln=10&seltype=2&uid=cl28563) | Predicted transcriptional regulator, containsd two HTH domains | 8-56 | 8.30E-03 |  |
| 46 | [DNA ligase](https://blast.ncbi.nlm.nih.gov/Blast.cgi#alnHdr_YP_009888457) | \|-\|28461\|29885 | 30 super family | [cl33690](https://www.ncbi.nlm.nih.gov/Structure/cdd/cddsrv.cgi?ascbin=8&maxaln=10&seltype=2&uid=cl33690) | DNA ligase; Provisional | 3-458 | 9.92E-152 |  |
| 47 | [1-acyl-sn-glycerol-3-phosphate acyltransferase](https://blast.ncbi.nlm.nih.gov/Blast.cgi#alnHdr_NQU54848) | \|-\|29942\|30109 |  |  |  |  |  |  |
| 48 | [hypothetical protein](https://blast.ncbi.nlm.nih.gov/Blast.cgi#alnHdr_QFR58386) | \|-\|30106\|30684 |  |  |  |  |  |  |
| 49 | [hypothetical protein](https://blast.ncbi.nlm.nih.gov/Blast.cgi#alnHdr_YP_009877648) | \|-\|30722\|31330 | PspA_IM30 super family | [cl37531](https://www.ncbi.nlm.nih.gov/Structure/cdd/cddsrv.cgi?ascbin=8&maxaln=10&seltype=2&uid=cl37531) | PspA/IM30 family; | 7-147 | 1.37E-04 |  |
| 50 | [DNA ligase](https://blast.ncbi.nlm.nih.gov/Blast.cgi#alnHdr_YP_009889065) | \|-\|31346\|31687 |  |  |  |  |  |  |
| 51 | [DNA ligase](https://blast.ncbi.nlm.nih.gov/Blast.cgi#alnHdr_AXF41613) | \|-\|31689\|31898 |  |  |  |  |  |  |
| 52 | [hypothetical protein](https://blast.ncbi.nlm.nih.gov/Blast.cgi#alnHdr_YP_009876082) | \|-\|31898\|32098 |  |  |  |  |  |  |
| 53 | [HNH endonuclease](https://blast.ncbi.nlm.nih.gov/Blast.cgi#alnHdr_ECW1086613) | \|-\|32098\|32214 |  |  |  |  |  |  |
| 54 | [hypothetical protein](https://blast.ncbi.nlm.nih.gov/Blast.cgi#alnHdr_YP_009283860) | \|-\|32211\|33404 |  |  |  |  |  |  |
| 55 | [hypothetical protein](https://blast.ncbi.nlm.nih.gov/Blast.cgi#alnHdr_YP_009140293) | \|-\|33522\|33824 |  |  |  |  |  |  |
| 56 | [hypothetical protein](https://blast.ncbi.nlm.nih.gov/Blast.cgi#alnHdr_YP_009220956) | \|-\|33837\|34169 |  |  |  |  |  |  |
| 57 | [DNA primase-helicase subunit](https://blast.ncbi.nlm.nih.gov/Blast.cgi#alnHdr_YP_007002692) | \|-\|34233\|35657 | 41 super family | [cl29348](https://www.ncbi.nlm.nih.gov/Structure/cdd/cddsrv.cgi?ascbin=8&maxaln=10&seltype=2&uid=cl29348) | 41 helicase; Provisional | 3-471 | 0.00E+00 |  |
| 58 | [hypothetical protein](https://blast.ncbi.nlm.nih.gov/Blast.cgi#alnHdr_YP_009021312) | \|-\|35664\|35993 |  |  |  |  |  |  |
| 59 | [RecA-like recombination protein](https://blast.ncbi.nlm.nih.gov/Blast.cgi#alnHdr_YP_009887835) | \|-\|35971\|37056 | RecA | [COG0468](https://www.ncbi.nlm.nih.gov/Structure/cdd/cddsrv.cgi?ascbin=8&maxaln=10&seltype=2&uid=COG0468) | RecA/RadA recombinase [Replication, recombination and repair]; | 36-272 | 8.56E-08 |  |
| 60 | [hypothetical protein](https://blast.ncbi.nlm.nih.gov/Blast.cgi#alnHdr_AYJ73567) | \|-\|37041\|37571 |  |  |  |  |  |  |
| 61 | [dUTPase](https://blast.ncbi.nlm.nih.gov/Blast.cgi#alnHdr_YP_007008025) | \|-\|37571\|38122 | NTP-PPase super family | [cl16941](https://www.ncbi.nlm.nih.gov/Structure/cdd/cddsrv.cgi?ascbin=8&maxaln=10&seltype=2&uid=cl16941) | Nucleoside Triphosphate Pyrophosphohydrolase (EC 3.6.1.8) MazG-like domain superfamily | 7-123 | 4.52E-05 |  |
| 62 | [putative deoxynucleotide monophosphate kinase](https://blast.ncbi.nlm.nih.gov/Blast.cgi#alnHdr_YP_009888237) | \|-\|38119\|38688 |  |  |  |  |  |  |
| 63 | [thymidylate synthase](https://blast.ncbi.nlm.nih.gov/Blast.cgi#alnHdr_YP_009887831) | \|-\|38685\|39740 | TS_Pyrimidine_HMase super family | [cl19097](https://www.ncbi.nlm.nih.gov/Structure/cdd/cddsrv.cgi?ascbin=8&maxaln=10&seltype=2&uid=cl19097) | Thymidylate synthase and pyrimidine hydroxymethylase | 12-229 | 4.43E-30 |  |
| 64 | [putative kinase/baseplate hub](https://blast.ncbi.nlm.nih.gov/Blast.cgi#alnHdr_YP_009140284) | \|-\|39740\|40402 | Ploopntkinase1 | [pfam18748](https://www.ncbi.nlm.nih.gov/Structure/cdd/cddsrv.cgi?ascbin=8&maxaln=10&seltype=2&uid=pfam18748) | P-loop Nucleotide Kinase1 | 14-209 | 1.72E-135 |  |
| 65 | [hypothetical protein](https://blast.ncbi.nlm.nih.gov/Blast.cgi#alnHdr_YP_009140283) | \|-\|40475\|41434 | PTZ00121 super family | [cl31754](https://www.ncbi.nlm.nih.gov/Structure/cdd/cddsrv.cgi?ascbin=8&maxaln=10&seltype=2&uid=cl31754) | MAEBL; Provisional | 36-214 | 9.50E-03 |  |
| 66 | [hypothetical protein](https://blast.ncbi.nlm.nih.gov/Blast.cgi#alnHdr_EDL0981964) | \|-\|41608\|41895 |  |  |  |  |  |  |
| 67 | [hypothetical protein](https://blast.ncbi.nlm.nih.gov/Blast.cgi#alnHdr_AXF41598) | \|-\|41913\|42659 |  |  |  |  |  |  |
| 68 | [DNA end protector protein](https://blast.ncbi.nlm.nih.gov/Blast.cgi#alnHdr_ANT44558) | \|-\|42721\|43425 | 2 | [PHA02577](https://www.ncbi.nlm.nih.gov/Structure/cdd/cddsrv.cgi?ascbin=8&maxaln=10&seltype=2&uid=PHA02577) | DNA end protector protein; Provisional | 36-233 | 2.17E-67 |  |
| 69 | [baseplate tail tube](https://blast.ncbi.nlm.nih.gov/Blast.cgi#alnHdr_YP_007008033) | \|+\|43480\|44421 |  |  |  |  |  |  |
| 70 | [ssDNA binding protein](https://blast.ncbi.nlm.nih.gov/Blast.cgi#alnHdr_YP_007008034) | \|-\|44449\|45480 | gp32 super family | [cl17537](https://www.ncbi.nlm.nih.gov/Structure/cdd/cddsrv.cgi?ascbin=8&maxaln=10&seltype=2&uid=cl17537) | gp32 DNA binding protein like | 3-342 | 6.12E-102 |  |
| 71 | [hypothetical protein](https://blast.ncbi.nlm.nih.gov/Blast.cgi#alnHdr_YP_009220936) | \|-\|45577\|45816 |  |  |  |  |  |  |
| 72 | [late promoter transcription factor](https://blast.ncbi.nlm.nih.gov/Blast.cgi#alnHdr_YP_007008035) | \|-\|45826\|46068 | Trans_coact super family | [cl25123](https://www.ncbi.nlm.nih.gov/Structure/cdd/cddsrv.cgi?ascbin=8&maxaln=10&seltype=2&uid=cl25123) | Phage late-transcription coactivator | 12-80 | 1.44E-19 |  |
| 73 | [regulatory protein](https://blast.ncbi.nlm.nih.gov/Blast.cgi#alnHdr_YP_007008036) | \|-\|46061\|46306 | Zn-ribbon_8 super family | [cl00993](https://www.ncbi.nlm.nih.gov/Structure/cdd/cddsrv.cgi?ascbin=8&maxaln=10&seltype=2&uid=cl00993) | Zinc ribbon domain; | 1-38 | 5.48E-07 |  |
| 74 | [hypothetical protein](https://blast.ncbi.nlm.nih.gov/Blast.cgi#alnHdr_YP_009617653) | \|-\|46293\|46589 |  |  |  |  |  |  |
| 75 | [RuvC-like endodeoxyribonuclease](https://blast.ncbi.nlm.nih.gov/Blast.cgi#alnHdr_YP_009798801) | \|-\|46582\|47193 |  |  |  |  |  |  |
| 76 | [hypothetical protein](https://blast.ncbi.nlm.nih.gov/Blast.cgi#alnHdr_YP_008770960) | \|-\|47231\|47716 |  |  |  |  |  |  |
| 77 | [hypothetical protein](https://blast.ncbi.nlm.nih.gov/Blast.cgi#alnHdr_YP_009880209) | \|-\|47694\|48218 |  |  |  |  |  |  |
| 78 | [baseplate hub subunit](https://blast.ncbi.nlm.nih.gov/Blast.cgi#alnHdr_YP_009888221) | \|+\|48268\|49074 | T4_baseplate super family | [cl27821](https://www.ncbi.nlm.nih.gov/Structure/cdd/cddsrv.cgi?ascbin=8&maxaln=10&seltype=2&uid=cl27821) | T4 bacteriophage base plate | 118-267 | 5.76E-08 |  |
| 79 | [baseplate hub subunit and tail lysozyme](https://blast.ncbi.nlm.nih.gov/Blast.cgi#alnHdr_YP_009888018) | \|+\|49581\|51206 | 5 super family | [cl33691](https://www.ncbi.nlm.nih.gov/Structure/cdd/cddsrv.cgi?ascbin=8&maxaln=10&seltype=2&uid=cl33691) | baseplate hub subunit and tail lysozyme; Provisional | 3-441 | 4.09E-39 |  |
| 80 | [baseplate wedge subunit](https://blast.ncbi.nlm.nih.gov/Blast.cgi#alnHdr_YP_009890073) | \|+\|51271\|51651 | GPW_gp25 super family | [cl01403](https://www.ncbi.nlm.nih.gov/Structure/cdd/cddsrv.cgi?ascbin=8&maxaln=10&seltype=2&uid=cl01403) | Gene 25-like lysozym | 4-126 | 6.78E-26 |  |
| 81 | [hypothetical protein](https://blast.ncbi.nlm.nih.gov/Blast.cgi#alnHdr_YP_009889036) | \|-\|51652\|51990 |  |  |  |  |  |  |
| 82 | [hypothetical protein](https://blast.ncbi.nlm.nih.gov/Blast.cgi#alnHdr_YP_009101407) | \|-\|51992\|52453 |  |  |  |  |  |  |
| 83 | [hypothetical protein](https://blast.ncbi.nlm.nih.gov/Blast.cgi#alnHdr_YP_009021295) | \|-\|52455\|52688 |  |  |  |  |  |  |
| 84 | [Glutaredoxin](https://blast.ncbi.nlm.nih.gov/Blast.cgi#alnHdr_YP_004327452) | \|-\|52795\|53019 | Thioredoxin_like super family | [cl00388](https://www.ncbi.nlm.nih.gov/Structure/cdd/cddsrv.cgi?ascbin=8&maxaln=10&seltype=2&uid=cl00388) | Protein Disulfide Oxidoreductases and Other Proteins with a Thioredoxin fold | 1-63 | 1.98E-07 |  |
| 85 | [ribonucleotide-diphosphate reductase beta subunit](https://blast.ncbi.nlm.nih.gov/Blast.cgi#alnHdr_YP_009879763) | \|-\|53029\|54132 | Ferritin_like super family | [cl00264](https://www.ncbi.nlm.nih.gov/Structure/cdd/cddsrv.cgi?ascbin=8&maxaln=10&seltype=2&uid=cl00264) | Ferritin-like superfamily of diiron-containing four-helix-bundle proteins | 21-362 | 1.19E-131 |  |
| 86 | [ribonucleoside-diphosphate reductase](https://blast.ncbi.nlm.nih.gov/Blast.cgi#alnHdr_QKE54727) | \|-\|54203\|56479 | nrdA super family | [cl33688](https://www.ncbi.nlm.nih.gov/Structure/cdd/cddsrv.cgi?ascbin=8&maxaln=10&seltype=2&uid=cl33688) | ribonucleoside-diphosphate reductase subunit alpha; Provisional | 1-758 | 0.00E+00 |  |
| 87 | [PhoH-like phosphate starvation-inducible protein](https://blast.ncbi.nlm.nih.gov/Blast.cgi#alnHdr_YP_004327456) | \|-\|56564\|57403 | P-loop_NTPase super family | [cl38936](https://www.ncbi.nlm.nih.gov/Structure/cdd/cddsrv.cgi?ascbin=8&maxaln=10&seltype=2&uid=cl38936) | P-loop containing Nucleoside Triphosphate Hydrolases | 45-222 | 3.50E-43 |  |
| 88 | [endolysin](https://blast.ncbi.nlm.nih.gov/Blast.cgi#alnHdr_YP_009879759) | \|-\|57511\|58305 | Muraidase | [pfam11860](https://www.ncbi.nlm.nih.gov/Structure/cdd/cddsrv.cgi?ascbin=8&maxaln=10&seltype=2&uid=pfam11860) | N-acetylmuramidase Endolysins | 90-263 | 6.57E-75 |  |
|  |  |  |  |  |  |  |  |  |
| 89 | [hypothetical protein](https://blast.ncbi.nlm.nih.gov/Blast.cgi#alnHdr_YP_009021287) | \|-\|58375\|58563 |  |  |  |  |  |  |
| 90 | [hypothetical protein](https://blast.ncbi.nlm.nih.gov/Blast.cgi#alnHdr_AXF41575) | \|-\|58614\|59102 |  |  |  |  |  |  |
| 91 | [hypothetical protein](https://blast.ncbi.nlm.nih.gov/Blast.cgi#alnHdr_YP_009021285) | \|-\|59302\|59511 |  |  |  |  |  |  |
| 92 | [DNA primase](https://blast.ncbi.nlm.nih.gov/Blast.cgi#alnHdr_QEA10245) | \|-\|59508\|60572 | 61 super family | [cl26791](https://www.ncbi.nlm.nih.gov/Structure/cdd/cddsrv.cgi?ascbin=8&maxaln=10&seltype=2&uid=cl26791) | DNA primase; Provisional | 3-354 | 5.28E-135 |  |
| 93 | [hypothetical protein](https://blast.ncbi.nlm.nih.gov/Blast.cgi#alnHdr_ECW1086572) | \|-\|60572\|61189 |  |  |  |  |  |  |
| 94 | [hypothetical protein](https://blast.ncbi.nlm.nih.gov/Blast.cgi#alnHdr_AYC62396) | \|-\|61253\|61606 | rI.-1 super family | [cl14362](https://www.ncbi.nlm.nih.gov/Structure/cdd/cddsrv.cgi?ascbin=8&maxaln=10&seltype=2&uid=cl14362) | hypothetical protein; Provisional | 7-113 | 1.60E-30 |  |
| 95 | [hypothetical protein](https://blast.ncbi.nlm.nih.gov/Blast.cgi#alnHdr_YP_009883213) | \|-\|61663\|62208 |  |  |  |  |  |  |
| 96 | [hypothetical protein](https://blast.ncbi.nlm.nih.gov/Blast.cgi#alnHdr_YP_009888610) | \|-\|62260\|64647 |  |  |  |  |  |  |
| 97 | [hypothetical protein](https://blast.ncbi.nlm.nih.gov/Blast.cgi#alnHdr_YP_009021278) | \|-\|64729\|65097 |  |  |  |  |  |  |
| 98 | [hypothetical protein](https://blast.ncbi.nlm.nih.gov/Blast.cgi#alnHdr_YP_009101589) | \|-\|65202\|65840 |  |  |  |  |  |  |
| 99 | [RegB site-specific RNA endonuclease/lytic transglycosylase](https://blast.ncbi.nlm.nih.gov/Blast.cgi#alnHdr_AXF41566) | \|-\|65840\|66457 |  |  |  |  |  |  |
| 100 | [hypothetical protein](https://blast.ncbi.nlm.nih.gov/Blast.cgi#alnHdr_YP_009021275) | \|-\|66554\|66838 |  |  |  |  |  |  |
| 101 | [hypothetical protein](https://blast.ncbi.nlm.nih.gov/Blast.cgi#alnHdr_YP_009881400) | \|-\|66819\|67148 | DUF4326 | [pfam14216](https://www.ncbi.nlm.nih.gov/Structure/cdd/cddsrv.cgi?ascbin=8&maxaln=10&seltype=2&uid=pfam14216) | Domain of unknown | 2-78 | 1.40E-27 |  |
| 102 | [hypothetical protein](https://blast.ncbi.nlm.nih.gov/Blast.cgi#alnHdr_YP_009889414) | \|-\|67145\|67453 | PHA02092 super family | [cl10367](https://www.ncbi.nlm.nih.gov/Structure/cdd/cddsrv.cgi?ascbin=8&maxaln=10&seltype=2&uid=cl10367) | hypothetical protein | 2-99 | 4.98E-05 |  |
| 103 | [recombination endonuclease subunit](https://blast.ncbi.nlm.nih.gov/Blast.cgi#alnHdr_YP_009888603) | \|-\|67457\|69790 | 46 super family | [cl33686](https://www.ncbi.nlm.nih.gov/Structure/cdd/cddsrv.cgi?ascbin=8&maxaln=10&seltype=2&uid=cl33686) | endonuclease subunit; Provisional | 36-760 | 4.52E-70 |  |
| 104 | [recombination protein subunit/recombination endonucelase subunit](https://blast.ncbi.nlm.nih.gov/Blast.cgi#alnHdr_YP_009030522) | \|-\|69793\|70908 | 47 super family | [cl26377](https://www.ncbi.nlm.nih.gov/Structure/cdd/cddsrv.cgi?ascbin=8&maxaln=10&seltype=2&uid=cl26377) | endonuclease subunit; Provisional | 1-347 | 1.65E-67 |  |
| 105 | [transcription sigma factor](https://blast.ncbi.nlm.nih.gov/Blast.cgi#alnHdr_YP_009879742) | \|-\|70895\|71686 | 55 super family | [cl14345](https://www.ncbi.nlm.nih.gov/Structure/cdd/cddsrv.cgi?ascbin=8&maxaln=10&seltype=2&uid=cl14345) | RNA polymerase sigma factor; Provisional | 20-186 | 9.34E-11 |  |
| 106 | [RNaseH](https://blast.ncbi.nlm.nih.gov/Blast.cgi#alnHdr_YP_009881842) | \|-\|71698\|72225 | RNase_HI_prokaryote_like | [cd09278](https://www.ncbi.nlm.nih.gov/Structure/cdd/cddsrv.cgi?ascbin=8&maxaln=10&seltype=2&uid=cd09278) | RNase HI family found mainly in prokaryotes; | 1-147 | 2.58E-36 |  |
| 107 | [hypothetical protein](https://blast.ncbi.nlm.nih.gov/Blast.cgi#alnHdr_YP_009881841) | \|+\|72268\|73035 |  |  |  |  |  |  |
| 108 | [ATP-dependent helicase](https://blast.ncbi.nlm.nih.gov/Blast.cgi#alnHdr_YP_009877708) | \|-\|73032\|74753 | DinG super family | [cl34176](https://www.ncbi.nlm.nih.gov/Structure/cdd/cddsrv.cgi?ascbin=8&maxaln=10&seltype=2&uid=cl34176) | Rad3-related DNA helicase [Replication, recombination and repair]; | 20-553 | 2.99E-31 |  |
| 109 | [DNA-binding protein HU-beta](https://blast.ncbi.nlm.nih.gov/Blast.cgi#alnHdr_YP_009887406) | \|-\|74889\|75167 | HU | [cd13831](https://www.ncbi.nlm.nih.gov/Structure/cdd/cddsrv.cgi?ascbin=8&maxaln=10&seltype=2&uid=cd13831) | histone-like DNA-binding protein HU | 2-86 | 1.01E-31 |  |
| 110 | [hypothetical protein](https://blast.ncbi.nlm.nih.gov/Blast.cgi#alnHdr_ARB06294) | \|-\|75251\|76042 | SPFH_like super family | [cl19107](https://www.ncbi.nlm.nih.gov/Structure/cdd/cddsrv.cgi?ascbin=8&maxaln=10&seltype=2&uid=cl19107) | core domain of the SPFH (stomatin, prohibitin, flotillin, and HflK/C) superfamily | 24-216 | 6.63E-17 |  |
| 111 | [sensor histidine kinase](https://blast.ncbi.nlm.nih.gov/Blast.cgi#alnHdr_WP_041076378) | \|-\|76042\|76251 |  |  |  |  |  |  |
| 112 | [superinfection exclusion protein](https://blast.ncbi.nlm.nih.gov/Blast.cgi#alnHdr_YP_009888187) | \|-\|76248\|76568 | Gp17 super family | [cl10305](https://www.ncbi.nlm.nih.gov/Structure/cdd/cddsrv.cgi?ascbin=8&maxaln=10&seltype=2&uid=cl10305) | Superinfection exclusion protein, bacteriophage P22 | 8-101 | 3.25E-53 |  |
| 113 | [hypothetical protein](https://blast.ncbi.nlm.nih.gov/Blast.cgi#alnHdr_YP_007008069) | \|-\|76558\|76845 |  |  |  |  |  |  |
| 114 | [hypothetical protein](https://blast.ncbi.nlm.nih.gov/Blast.cgi#alnHdr_YP_009140231) | \|-\|76847\|77515 |  |  |  |  |  |  |
| 115 | [hypothetical protein](https://blast.ncbi.nlm.nih.gov/Blast.cgi#alnHdr_YP_009888184) | \|-\|77572\|78363 |  |  |  |  |  |  |
| 116 | [hypothetical protein](https://blast.ncbi.nlm.nih.gov/Blast.cgi#alnHdr_YP_009283923) | \|-\|78413\|78733 |  |  |  |  |  |  |
| 117 | [i-spanin](https://blast.ncbi.nlm.nih.gov/Blast.cgi#alnHdr_YP_009888384) | \|-\|78730\|79419 |  |  |  |  |  |  |
| 118 | [hypothetical protein](https://blast.ncbi.nlm.nih.gov/Blast.cgi#alnHdr_AYJ73626) | \|-\|79504\|79953 |  |  |  |  |  |  |
| 119 | [hypothetical protein](https://blast.ncbi.nlm.nih.gov/Blast.cgi#alnHdr_AGF89117) | \|-\|79985\|80152 |  |  |  |  |  |  |
| 120 | [hypothetical protein](https://blast.ncbi.nlm.nih.gov/Blast.cgi#alnHdr_YP_009888586) | \|-\|80154\|80438 |  |  |  |  |  |  |
| 121 | [hypothetical protein](https://blast.ncbi.nlm.nih.gov/Blast.cgi#alnHdr_YP_009888585) | \|-\|80449\|80961 |  |  |  |  |  |  |
| 122 | [hypothetical protein](https://blast.ncbi.nlm.nih.gov/Blast.cgi#alnHdr_AXY85202) | \|-\|81021\|81260 |  |  |  |  |  |  |
| 123 | [acyl carrier protein](https://blast.ncbi.nlm.nih.gov/Blast.cgi#alnHdr_YP_009888583) | \|-\|81387\|81698 | PP-binding super family | [cl09936](https://www.ncbi.nlm.nih.gov/Structure/cdd/cddsrv.cgi?ascbin=8&maxaln=10&seltype=2&uid=cl09936) | Phosphopantetheine | 54-80 | 5.14E-07 |  |
| 124 | [von Willebrand factor type A domain protein](https://blast.ncbi.nlm.nih.gov/Blast.cgi#alnHdr_AXF41739) | \|-\|81742\|83958 | vWFA super family | [cl00057](https://www.ncbi.nlm.nih.gov/Structure/cdd/cddsrv.cgi?ascbin=8&maxaln=10&seltype=2&uid=cl00057) | Von Willebrand factor type A (vWA) domain | 20-179 | 2.87E-08 |  |
| 125 | [hypothetical protein](https://blast.ncbi.nlm.nih.gov/Blast.cgi#alnHdr_YP_009030502) | \|-\|83951\|84121 |  |  |  |  |  |  |
| 126 | [hypothetical protein](https://blast.ncbi.nlm.nih.gov/Blast.cgi#alnHdr_YP_009101562) | \|-\|84123\|84482 |  |  |  |  |  |  |
| 127 | [hypothetical protein](https://blast.ncbi.nlm.nih.gov/Blast.cgi#alnHdr_YP_009875948) | \|-\|84582\|85307 | Phage_gp49_66 super family | [cl10351](https://www.ncbi.nlm.nih.gov/Structure/cdd/cddsrv.cgi?ascbin=8&maxaln=10&seltype=2&uid=cl10351) | Phage protein (N4 Gp49/phage Sf6 gene 66) family | 9-84 | 8.02E-14 |  |
| 128 | [hypothetical protein](https://blast.ncbi.nlm.nih.gov/Blast.cgi#alnHdr_YP_009879537) | \|-\|85300\|85551 |  |  |  |  |  |  |
| 129 | [hypothetical protein](https://blast.ncbi.nlm.nih.gov/Blast.cgi#alnHdr_YP_009879717) | \|-\|85523\|85933 | DUF3268 super family | [cl13172](https://www.ncbi.nlm.nih.gov/Structure/cdd/cddsrv.cgi?ascbin=8&maxaln=10&seltype=2&uid=cl13172) | zinc-finger-containing domain; | 2-120 | 1.59E-49 |  |
| 130 | [hypothetical protein](https://blast.ncbi.nlm.nih.gov/Blast.cgi#alnHdr_YP_009880156) | \|-\|85993\|86364 |  |  |  |  |  |  |
| 131 | [hypothetical protein](https://blast.ncbi.nlm.nih.gov/Blast.cgi#alnHdr_QFR58306) | \|-\|86403\|88424 | DUF2135 super family | [cl19870](https://www.ncbi.nlm.nih.gov/Structure/cdd/cddsrv.cgi?ascbin=8&maxaln=10&seltype=2&uid=cl19870) | Uncharacterized protein conserved in bacteria (DUF2135) | 411-480 | 1.04E-04 |  |
| 132 | [hypothetical protein](https://blast.ncbi.nlm.nih.gov/Blast.cgi#alnHdr_YP_009881819) | \|-\|88538\|89764 | aGPT-Pplase1 | [pfam18723](https://www.ncbi.nlm.nih.gov/Structure/cdd/cddsrv.cgi?ascbin=8&maxaln=10&seltype=2&uid=pfam18723) | alpha-glutamyl/putrescinyl thymine pyrophosphorylase clade 1 | 34-361 | 1.52E-101 |  |
| 133 | [hypothetical protein](https://blast.ncbi.nlm.nih.gov/Blast.cgi#alnHdr_YP_009881030) | \|-\|89834\|90697 | NTP-PPase super family | [cl16941](https://www.ncbi.nlm.nih.gov/Structure/cdd/cddsrv.cgi?ascbin=8&maxaln=10&seltype=2&uid=cl16941) | Nucleoside Triphosphate Pyrophosphohydrolase (EC 3.6.1.8) MazG-like domain superfamily | 14-114 | 2.28E-06 |  |
| 134 | [RegA translational repressor protein](https://blast.ncbi.nlm.nih.gov/Blast.cgi#alnHdr_YP_008532080) | \|-\|90714\|91178 | regA | [PHA02543](https://www.ncbi.nlm.nih.gov/Structure/cdd/cddsrv.cgi?ascbin=8&maxaln=10&seltype=2&uid=PHA02543) | translation repressor protein; Provisional | 21-148 | 3.29E-65 |  |
| 135 | [clamp holder for DNA polymerase](https://blast.ncbi.nlm.nih.gov/Blast.cgi#alnHdr_YP_009890021) | \|-\|91208\|91630 | Phage_clamp_A super family | [cl27778](https://www.ncbi.nlm.nih.gov/Structure/cdd/cddsrv.cgi?ascbin=8&maxaln=10&seltype=2&uid=cl27778) | Bacteriophage clamp loader A subunit | 3-106 | 2.29E-09 |  |
| 136 | [clamp loader small subunit](https://blast.ncbi.nlm.nih.gov/Blast.cgi#alnHdr_AGF89133) | \|-\|91635\|92624 | 44 super family | [cl33683](https://www.ncbi.nlm.nih.gov/Structure/cdd/cddsrv.cgi?ascbin=8&maxaln=10&seltype=2&uid=cl33683) | clamp loader, small subunit; Provisional | 3-329 | 7.92E-149 |  |
| 137 | [sliding clamp DNA polymerase accessory protein](https://blast.ncbi.nlm.nih.gov/Blast.cgi#alnHdr_YP_009101552) | \|-\|92703\|93371 | 45 super family | [cl31814](https://www.ncbi.nlm.nih.gov/Structure/cdd/cddsrv.cgi?ascbin=8&maxaln=10&seltype=2&uid=cl31814) | sliding clamp; Provisional | 6-222 | 2.51E-31 |  |
| 138 | [clamp loader subunit](https://blast.ncbi.nlm.nih.gov/Blast.cgi#alnHdr_YP_009140207) | \|+\|93711\|94076 | PTZ00491 super family | [cl36570](https://www.ncbi.nlm.nih.gov/Structure/cdd/cddsrv.cgi?ascbin=8&maxaln=10&seltype=2&uid=cl36570) | major vault protein; Provisional | 51-92 | 8.07E-03 |  |
| 139 | [DNA helicase](https://blast.ncbi.nlm.nih.gov/Blast.cgi#alnHdr_ARB06475) | \|-\|94082\|95581 | uvsW super family | [cl33684](https://www.ncbi.nlm.nih.gov/Structure/cdd/cddsrv.cgi?ascbin=8&maxaln=10&seltype=2&uid=cl33684) | UvsW helicase; Provisional | 1-499 | 0.00E+00 |  |
| 140 | [PD-(D/E)XK nuclease](https://blast.ncbi.nlm.nih.gov/Blast.cgi#alnHdr_YP_008770899) | \|-\|95611\|96357 | Cas4_I-A_I-B_I-C_I-D_II-B super family | [cl00641](https://www.ncbi.nlm.nih.gov/Structure/cdd/cddsrv.cgi?ascbin=8&maxaln=10&seltype=2&uid=cl00641) | CRISPR/Cas system-associated protein | 62-201 | 1.44E-05 |  |
| 141 | [DNA repair/recombination protein](https://blast.ncbi.nlm.nih.gov/Blast.cgi#alnHdr_YP_009030484) | \|-\|96357\|96812 | UvsY super family | [cl12619](https://www.ncbi.nlm.nih.gov/Structure/cdd/cddsrv.cgi?ascbin=8&maxaln=10&seltype=2&uid=cl12619) | Recombination, repair and ssDNA binding protein UvsY; | 30-151 | 2.12E-08 |  |
| 142 | [tail completion and sheath stabilizer](https://blast.ncbi.nlm.nih.gov/Blast.cgi#alnHdr_YP_007002776) | \|-\|96851\|97348 | 3 super family | [cl24094](https://www.ncbi.nlm.nih.gov/Structure/cdd/cddsrv.cgi?ascbin=8&maxaln=10&seltype=2&uid=cl24094) | tail completion and sheath stabilizer protein; Provisional | 12-150 | 1.29E-04 |  |
| 143 | [hypothetical protein](https://blast.ncbi.nlm.nih.gov/Blast.cgi#alnHdr_YP_009876502) | \|+\|97418\|98032 |  |  |  |  |  |  |
| 144 | [hypothetical protein](https://blast.ncbi.nlm.nih.gov/Blast.cgi#alnHdr_YP_009877743) | \|-\|98033\|98761 |  |  |  |  |  |  |
| 145 | [hypothetical protein](https://blast.ncbi.nlm.nih.gov/Blast.cgi#alnHdr_YP_009283951) | \|-\|98998\|99420 | DUF2493 super family | [cl15723](https://www.ncbi.nlm.nih.gov/Structure/cdd/cddsrv.cgi?ascbin=8&maxaln=10&seltype=2&uid=cl15723) | Protein of unknown function | 5-51 | 1.76E-07 |  |
| 146 | [hypothetical protein](https://blast.ncbi.nlm.nih.gov/Blast.cgi#alnHdr_YP_009030479) | \|-\|99426\|99743 |  |  |  |  |  |  |
| 147 | [hypothetical protein](https://blast.ncbi.nlm.nih.gov/Blast.cgi#alnHdr_YP_009875969) | \|-\|99812\|100276 |  |  |  |  |  |  |
| 148 | [hypothetical protein](https://blast.ncbi.nlm.nih.gov/Blast.cgi#alnHdr_YP_009889164) | \|-\|100380\|100616 |  |  |  |  |  |  |
| 149 | [hypothetical protein](https://blast.ncbi.nlm.nih.gov/Blast.cgi#alnHdr_ARB06463) | \|-\|100613\|100987 |  |  |  |  |  |  |
| 150 | [hypothetical protein](https://blast.ncbi.nlm.nih.gov/Blast.cgi#alnHdr_ARB06463) | \|-\|100996\|101436 | NBD_sugar-kinase_HSP70_actin super family | [cl17037](https://www.ncbi.nlm.nih.gov/Structure/cdd/cddsrv.cgi?ascbin=8&maxaln=10&seltype=2&uid=cl17037) | Nucleotide-Binding Domain of the sugar kinase/HSP70/actin superfamily | 86-134 | 3.53E-03 |  |
| 151 | [hypothetical protein](https://blast.ncbi.nlm.nih.gov/Blast.cgi#alnHdr_YP_009883267) | \|-\|101497\|102144 | GIY-YIG_SF super family | [cl15257](https://www.ncbi.nlm.nih.gov/Structure/cdd/cddsrv.cgi?ascbin=8&maxaln=10&seltype=2&uid=cl15257) | GIY-YIG nuclease domain superfamily; | 5-88 | 5.23E-11 |  |
| 152 | [major cpasid protein/major head protein](https://blast.ncbi.nlm.nih.gov/Blast.cgi#alnHdr_YP_007008101) | \|-\|102226\|103548 | Gp23 super family | [cl22495](https://www.ncbi.nlm.nih.gov/Structure/cdd/cddsrv.cgi?ascbin=8&maxaln=10&seltype=2&uid=cl22495) | Major capsid protein Gp23 | 7-439 | 5.03E-115 |  |
| 153 | [prohead core scaffold protein](https://blast.ncbi.nlm.nih.gov/Blast.cgi#alnHdr_YP_009881799) | \|-\|103640\|104473 | 22 super family | [cl20173](https://www.ncbi.nlm.nih.gov/Structure/cdd/cddsrv.cgi?ascbin=8&maxaln=10&seltype=2&uid=cl20173) | prohead core protein; Provisional | 4-213 | 8.95E-20 |  |
| 154 | [prohead core and protease](https://blast.ncbi.nlm.nih.gov/Blast.cgi#alnHdr_YP_009889159) | \|-\|104520\|105185 | Peptidase_S77 super family | [cl11614](https://www.ncbi.nlm.nih.gov/Structure/cdd/cddsrv.cgi?ascbin=8&maxaln=10&seltype=2&uid=cl11614) | Prohead core protein serine protease; | 24-175 | 4.31E-46 |  |
| 155 | [hypothetical protein](https://blast.ncbi.nlm.nih.gov/Blast.cgi#alnHdr_EBS2632373) | \|-\|105196\|105510 |  |  |  |  |  |  |
| 156 | [hypothetical protein](https://blast.ncbi.nlm.nih.gov/Blast.cgi#alnHdr_YP_009875981) | \|-\|105522\|105692 |  |  |  |  |  |  |
| 157 | [portal vertex protein of the head](https://blast.ncbi.nlm.nih.gov/Blast.cgi#alnHdr_YP_009881795) | \|-\|105734\|107416 | 20 | [PHA02531](https://www.ncbi.nlm.nih.gov/Structure/cdd/cddsrv.cgi?ascbin=8&maxaln=10&seltype=2&uid=PHA02531) | portal vertex protein; Provisional | 20-528 | 0.00E+00 |  |
| 158 | [tail tube protein](https://blast.ncbi.nlm.nih.gov/Blast.cgi#alnHdr_ARB06454) | \|-\|107484\|108017 | 19 super family | [cl28641](https://www.ncbi.nlm.nih.gov/Structure/cdd/cddsrv.cgi?ascbin=8&maxaln=10&seltype=2&uid=cl28641) | tail tube protein; Provisional | 1-168 | 4.05E-23 |  |
| 159 | [GIY-YIG endonuclease](https://blast.ncbi.nlm.nih.gov/Blast.cgi#alnHdr_YP_008770879) | \|-\|108048\|108506 | GIY-YIG_SF super family | [cl15257](https://www.ncbi.nlm.nih.gov/Structure/cdd/cddsrv.cgi?ascbin=8&maxaln=10&seltype=2&uid=cl15257) | GIY-YIG | 3-88 | 4.29E-14 |  |
| 160 | [tail sheath protein](https://blast.ncbi.nlm.nih.gov/Blast.cgi#alnHdr_YP_009876481) | \|-\|108564\|110459 | 18 super family | [cl33682](https://www.ncbi.nlm.nih.gov/Structure/cdd/cddsrv.cgi?ascbin=8&maxaln=10&seltype=2&uid=cl33682) | tail sheath protein; | 1-621 | 1.01E-99 |  |
| 161 | [terminase large subunit](https://blast.ncbi.nlm.nih.gov/Blast.cgi#alnHdr_YP_009876480) | \|-\|110512\|112722 | 17 super family | [cl28557](https://www.ncbi.nlm.nih.gov/Structure/cdd/cddsrv.cgi?ascbin=8&maxaln=10&seltype=2&uid=cl28557) | large terminase protein | 23-729 | 0.00E+00 |  |
| 162 | [terminase DNA packaging enzyme small subunit](https://blast.ncbi.nlm.nih.gov/Blast.cgi#alnHdr_YP_009887541) | \|-\|112703\|113383 | DNA_Packaging super family | [cl27835](https://www.ncbi.nlm.nih.gov/Structure/cdd/cddsrv.cgi?ascbin=8&maxaln=10&seltype=2&uid=cl27835) | Terminase DNA packaging enzyme; | 36-214 | 5.93E-10 |  |
| 163 | [proximal tail sheath stabilization protein](https://blast.ncbi.nlm.nih.gov/Blast.cgi#alnHdr_YP_009293354) | \|-\|113386\|114084 | T4-gp15_tss super family | [cl14348](https://www.ncbi.nlm.nih.gov/Structure/cdd/cddsrv.cgi?ascbin=8&maxaln=10&seltype=2&uid=cl14348) | T4-like virus Myoviridae tail sheath stabilizer | 8-210 | 2.94E-18 |  |
| 164 | [neck protein](https://blast.ncbi.nlm.nih.gov/Blast.cgi#alnHdr_YP_009879676) | \|-\|114087\|114728 | T4_neck-protein super family | [cl27828](https://www.ncbi.nlm.nih.gov/Structure/cdd/cddsrv.cgi?ascbin=8&maxaln=10&seltype=2&uid=cl27828) | Virus neck protein | 1-171 | 1.61E-71 |  |
| 165 | [hypothetical protein](https://blast.ncbi.nlm.nih.gov/Blast.cgi#alnHdr_YP_009876476) | \|+\|114790\|115002 |  |  |  |  |  |  |
| 166 | [neck protein](https://blast.ncbi.nlm.nih.gov/Blast.cgi#alnHdr_YP_007008113) | \|-\|115031\|115783 | 13 super family | [cl14347](https://www.ncbi.nlm.nih.gov/Structure/cdd/cddsrv.cgi?ascbin=8&maxaln=10&seltype=2&uid=cl14347) | neck protein | 4-226 | 2.27E-32 |  |
| 167 | [hypothetical protein](https://blast.ncbi.nlm.nih.gov/Blast.cgi#alnHdr_YP_009888944) | \|-\|115770\|116111 |  |  |  |  |  |  |
| 168 | [hypothetical protein](https://blast.ncbi.nlm.nih.gov/Blast.cgi#alnHdr_YP_009875993) | \|-\|116095\|116343 |  |  |  |  |  |  |
| 169 | [hypothetical protein](https://blast.ncbi.nlm.nih.gov/Blast.cgi#alnHdr_YP_009879670) | \|-\|116396\|121234 | DUF4815 super family | [cl24594](https://www.ncbi.nlm.nih.gov/Structure/cdd/cddsrv.cgi?ascbin=8&maxaln=10&seltype=2&uid=cl24594) | Domain of unknown function (DUF4815); | 11-929 | 4.57E-41 |  |
| 170 | [tailspike protein](https://blast.ncbi.nlm.nih.gov/Blast.cgi#alnHdr_YP_009877767) | \|-\|121326\|123233 | Tail_spike_N super family | [cl39985](https://www.ncbi.nlm.nih.gov/Structure/cdd/cddsrv.cgi?ascbin=8&maxaln=10&seltype=2&uid=cl39985) | Tail spike TSP1/Gp66 receptor binding N-terminal domain | 88-152 | 1.35E-13 |  |
| 171 | [tail fiber protein](https://blast.ncbi.nlm.nih.gov/Blast.cgi#alnHdr_AYJ73681) | \|-\|123281\|125524 | phage_tailspike_middle super family | [cl40411](https://www.ncbi.nlm.nih.gov/Structure/cdd/cddsrv.cgi?ascbin=8&maxaln=10&seltype=2&uid=cl40411) | N-terminal and middle domains bacteriophages | 291-428 | 3.92E-03 |  |
| 172 | [tailspike protein](https://blast.ncbi.nlm.nih.gov/Blast.cgi#alnHdr_AXY85148) | \|-\|125625\|127721 | PhageP22-tail super family | [cl07762](https://www.ncbi.nlm.nih.gov/Structure/cdd/cddsrv.cgi?ascbin=8&maxaln=10&seltype=2&uid=cl07762) | Salmonella phage P22 tail-spike | 166-692 | 9.58E-89 |  |
|  |  |  |  |  |  |  |  |  |
| 173 | [tailspike/hemolysin-type calcium-binding protein](https://blast.ncbi.nlm.nih.gov/Blast.cgi#alnHdr_YP_008770865) | \|-\|127772\|130945 | Beta_helix | [pfam13229](https://www.ncbi.nlm.nih.gov/Structure/cdd/cddsrv.cgi?ascbin=8&maxaln=10&seltype=2&uid=pfam13229) | Right handed beta helix regio | 625-739 | 3.72E-04 |  |
| 174 | [hypothetical protein](https://blast.ncbi.nlm.nih.gov/Blast.cgi#alnHdr_YP_009881974) | \|-\|130992\|132182 |  |  |  |  |  |  |
| 175 | [hypothetical protein](https://blast.ncbi.nlm.nih.gov/Blast.cgi#alnHdr_ECW1086689) | \|-\|132185\|133039 |  |  |  |  |  |  |
| 176 | [baseplate wedge subunit](https://blast.ncbi.nlm.nih.gov/Blast.cgi#alnHdr_YP_009283780) | \|-\|133023\|134804 | Baseplate_J super family | [cl01294](https://www.ncbi.nlm.nih.gov/Structure/cdd/cddsrv.cgi?ascbin=8&maxaln=10&seltype=2&uid=cl01294) | Baseplate J-like protein | 1-593 | 0.00E+00 |  |
| 177 | [hypothetical protein](https://blast.ncbi.nlm.nih.gov/Blast.cgi#alnHdr_YP_009283781) | \|+\|135168\|135737 |  |  |  |  |  |  |
| 178 | [hypothetical protein](https://blast.ncbi.nlm.nih.gov/Blast.cgi#alnHdr_YP_009881969) | \|+\|138166\|138366 |  |  |  |  |  |  |
| 179 | [hypothetical protein](https://blast.ncbi.nlm.nih.gov/Blast.cgi#alnHdr_QIQ62272) | \|+\|139361\|139594 |  |  |  |  |  |  |
| 180 | [tail completion protein](https://blast.ncbi.nlm.nih.gov/Blast.cgi#alnHdr_YP_009884719) | \|+\|139604\|140122 | Phage_tail_NK super family | [cl24937](https://www.ncbi.nlm.nih.gov/Structure/cdd/cddsrv.cgi?ascbin=8&maxaln=10&seltype=2&uid=cl24937) | Sf6-type phage tail needle knob or tip of some Caudovirales | 15-163 | 8.40E-30 |  |
| 181 | [hypothetical protein](https://blast.ncbi.nlm.nih.gov/Blast.cgi#alnHdr_YP_009888930) | \|+\|140194\|140880 | ETC_C1_NDUFA4 super family | [cl04760](https://www.ncbi.nlm.nih.gov/Structure/cdd/cddsrv.cgi?ascbin=8&maxaln=10&seltype=2&uid=cl04760) | ETC complex I subunit conserved region | 72-132 | 5.28E-04 |  |
| 182 | [hypothetical protein](https://blast.ncbi.nlm.nih.gov/Blast.cgi#alnHdr_YP_009879594) | \|+\|140950\|142143 | PALP super family | [cl29017](https://www.ncbi.nlm.nih.gov/Structure/cdd/cddsrv.cgi?ascbin=8&maxaln=10&seltype=2&uid=cl29017) | Pyridoxal-phosphate dependent enzyme | 26-245 | 6.47E-08 |  |
| 183 | [hypothetical protein](https://blast.ncbi.nlm.nih.gov/Blast.cgi#alnHdr_YP_009030439) | \|+\|142183\|142632 | ART-PolyVal super family | [cl40065](https://www.ncbi.nlm.nih.gov/Structure/cdd/cddsrv.cgi?ascbin=8&maxaln=10&seltype=2&uid=cl40065) | ADP-Ribosyltransferase in polyvalent proteins | 20-143 | 1.04E-08 |  |
| 184 | [hypothetical protein](https://blast.ncbi.nlm.nih.gov/Blast.cgi#alnHdr_YP_009140366) | \|+\|142668\|143126 |  |  |  |  |  |  |
| 185 | [hypothetical protein](https://blast.ncbi.nlm.nih.gov/Blast.cgi#alnHdr_YP_009888926) | \|-\|143153\|143527 |  |  |  |  |  |  |
| 186 | [hypothetical protein](https://blast.ncbi.nlm.nih.gov/Blast.cgi#alnHdr_YP_009293379) | \|-\|143524\|144066 |  |  |  |  |  |  |
| 187 | [hypothetical protein](https://blast.ncbi.nlm.nih.gov/Blast.cgi#alnHdr_YP_007002826) | \|-\|144115\|144672 |  |  |  |  |  |  |
| 188 | [hypothetical protein](https://blast.ncbi.nlm.nih.gov/Blast.cgi#alnHdr_EBS2632543) | \|-\|144768\|144980 |  |  |  |  |  |  |
| 189 | [hypothetical protein](https://blast.ncbi.nlm.nih.gov/Blast.cgi#alnHdr_YP_007002828) | \|-\|145022\|145612 |  |  |  |  |  |  |
| 190 | [DNA polymerase](https://blast.ncbi.nlm.nih.gov/Blast.cgi#alnHdr_AYJ73701) | \|+\|145674\|148670 | 43 super family | [cl31813](https://www.ncbi.nlm.nih.gov/Structure/cdd/cddsrv.cgi?ascbin=8&maxaln=10&seltype=2&uid=cl31813) | DNA polymerase; Provisional | 1-995 | 0.00E+00 |  |
| 191 | [hypothetical protein](https://blast.ncbi.nlm.nih.gov/Blast.cgi#alnHdr_YP_009293385) | \|+\|148731\|149075 |  |  |  |  |  |  |
| 192 | [5'(3')-deoxyribonucleotidase](https://blast.ncbi.nlm.nih.gov/Blast.cgi#alnHdr_YP_008770845) | \|+\|149068\|149877 | HAD_like super family | [cl21460](https://www.ncbi.nlm.nih.gov/Structure/cdd/cddsrv.cgi?ascbin=8&maxaln=10&seltype=2&uid=cl21460) | Haloacid | 19-201 | 6.22E-06 |  |
| 193 | [hypothetical protein](https://blast.ncbi.nlm.nih.gov/Blast.cgi#alnHdr_YP_009876443) | \|+\|149861\|150166 |  |  |  |  |  |  |
| 194 | [hypothetical protein](https://blast.ncbi.nlm.nih.gov/Blast.cgi#alnHdr_YP_009877795) | \|+\|150166\|151071 | Ploopntkinase2 super family | [cl40054](https://www.ncbi.nlm.nih.gov/Structure/cdd/cddsrv.cgi?ascbin=8&maxaln=10&seltype=2&uid=cl40054) | P-loop Nucleotide Kinase2 | 1-289 | 8.86E-177 |  |
| 195 | [hypothetical protein](https://blast.ncbi.nlm.nih.gov/Blast.cgi#alnHdr_YP_009140355) | \|+\|151074\|151277 |  |  |  |  |  |  |
| 196 | [hypothetical protein](https://blast.ncbi.nlm.nih.gov/Blast.cgi#alnHdr_YP_009888509) | \|+\|151279\|151629 |  |  |  |  |  |  |
| 197 | [hypothetical protein](https://blast.ncbi.nlm.nih.gov/Blast.cgi#alnHdr_YP_009876027) | \|+\|151613\|152857 | aGPT-Pplase2 | [pfam18724](https://www.ncbi.nlm.nih.gov/Structure/cdd/cddsrv.cgi?ascbin=8&maxaln=10&seltype=2&uid=pfam18724) | Alpha-glutamyl/putrescinyl thymine pyrophosphorylase clade 2 | 48-292 | 3.72E-49 |  |
| 198 | [hypothetical protein](https://blast.ncbi.nlm.nih.gov/Blast.cgi#alnHdr_YP_007236276) | \|+\|152893\|153060 |  |  |  |  |  |  |
| 199 | [hypothetical protein](https://blast.ncbi.nlm.nih.gov/Blast.cgi#alnHdr_YP_009887707) | \|+\|153143\|153364 |  |  |  |  |  |  |
| 200 | [hypothetical protein](https://blast.ncbi.nlm.nih.gov/Blast.cgi#alnHdr_YP_009887706) | \|+\|153361\|153747 |  |  |  |  |  |  |
| 201 | [hypothetical protein](https://blast.ncbi.nlm.nih.gov/Blast.cgi#alnHdr_YP_009140348) | \|+\|153744\|154853 | TerC super family | [cl10468](https://www.ncbi.nlm.nih.gov/Structure/cdd/cddsrv.cgi?ascbin=8&maxaln=10&seltype=2&uid=cl10468) | Integral membrane protein | 2-69 | 1.80E-03 |  |
| 202 | [hypothetical protein](https://blast.ncbi.nlm.nih.gov/Blast.cgi#alnHdr_YP_009879629) | \|+\|154869\|155438 | HAD_like super family | [cl21460](https://www.ncbi.nlm.nih.gov/Structure/cdd/cddsrv.cgi?ascbin=8&maxaln=10&seltype=2&uid=cl21460) | Haloacid Dehalogenase-like Hydrolases | 4-53 | 4.48E-03 |  |
|  |  |  |  |  |  |  |  |  |
| 203 | [hypothetical protein](https://blast.ncbi.nlm.nih.gov/Blast.cgi#alnHdr_YP_009140346) | \|+\|155443\|155571 |  |  |  |  |  |  |
| 204 | [hypothetical protein](https://blast.ncbi.nlm.nih.gov/Blast.cgi#alnHdr_YP_009140345) | \|+\|155571\|155996 |  |  |  |  |  |  |
